# Neutrophil extracellular traps and macrophage activation contibute to thrombosis and post-covid syndrome in SARS-CoV-2 infection

**DOI:** 10.3389/fimmu.2025.1507167

**Published:** 2025-02-24

**Authors:** Irene Serrano-Gonzalo, Bárbara Menéndez-Jandula, Esther Franco-García, Isidro Arévalo-Vargas, Calos Lahoz-Gil, Paz Latre, Sonia Roca-Esteve, Ralf Köhler, Laura López de Frutos, Pilar Giraldo

**Affiliations:** ^1^ Fundación Española para el Estudio y Terapéutica de la Enfermedad de Gaucher y otras lisosomales (FEETEG), Zaragoza, Spain; ^2^ Grupo de Investigación Mecanismos de Enfermedad Crónica e Investigación Traslacional (MECIT), Zaragoza, Spain; ^3^ Grupo Estudio de Enfermedades de Depósito Lisosomal (GEEDL), Sociedad Española de Hematología y Hemoterapia (SEHH), Zaragoza, Spain; ^4^ Grupo de Investigación en Enfermedad de Gaucher (GIIS-012), Instituto de Investigación Sanitaria Aragón, Zaragoza, Spain; ^5^ Servicio de Hematología, Hospital Universitario Miguel Servet., Zaragoza, Spain; ^6^ Servicio de Hematología, Hospital Ntra Sra de Gracia, Zaragoza, Spain; ^7^ Medicina de Familia, Servicio de Atención primaria., Zaragoza, Spain; ^8^ Fundación Aragonesa para la Investigación y el Desarrollo (ARAID), Zaragoza, Spain; ^9^ Servicio de Hematología, Hospital QuironSalud, Zaragoza, Spain

**Keywords:** SARS-CoV-2, neutrophils extracellular traps (NETs), thrombosis, post-COVID syndrome, macrophage biomarkers

## Abstract

**Background:**

SARS-CoV-2 infection activates macrophages and induces the release of neutrophil extracellular traps (NETs). Excess NETs is linked to inflammatory and thrombotic complications observed in COVID-19.

**Aim:**

To explore the impact of NETs and macrophage activation on SARS-CoV-2-infected patients who developed complications.

**Methods:**

We included 30 patients from the first (March 2020) and 30 from the second wave (July 2021), collecting two plasma samples at diagnosis and seven days later. Data on demographics, comorbidities, and basic analytical data were compiled. NETs markers (myeloperoxidase (MPO), neutrophil elastase (NE), p-selectin (P-SEL) and S100A8/S100A9 heterodimer (MRP)) and macrophage activation markers (Chitotriosidase activity (ChT), CCL18/PARC and YKL-40) were measured.

**Results:**

The first wave had higher incidences of post-COVID syndrome, ICU admissions, and mortality. Patients of each wave showed elevated blood cells, liver enzymes, and coagulation markers at the time of diagnosis, with fibrinogen and D-Dimer differing between waves. NET and macrophage markers, NE, MPO, MRP, DNAse, ChT, and CCL18 were elevated, while P-SEL, cfDNA, and YKL-40 were decreased if compared to controls. A decrease in NE and DNAse is a link to lower levels of these two markers in complications versus without complications.

**Conclusions:**

This study emonstrates alterations in NETs and macrophage activation markers in COVID-19 patients, indicating an imbalance in inflammatory response regulation.

## 1 Introduction

The COVID-19 pandemic caused by the coronavirus 2 (SARS-CoV-2) has led to significant morbidity and mortality worldwide and presents a new challenge to investigate the pathophysiology of the complications that occur with this infection (1–3). One of the major complications associated with this viral infection is the development of thrombotic events, which can affect various organ systems and contribute to disease severity (4–6). Understanding the dynamics of immune responses in COVID-19 patients is crucial for unraveling the complexities of the disease (7, 8). A dysregulated innate immune response has been observed (9), with the activation of neutrophils and macrophages—key components of the immune response—implicated in the pathogenesis of thrombotic events in COVID-19 patients (10).

The prevalence of post-COVID syndrome is estimated to be around 30% in the general population. This syndrome is characterized by a range of persistent signs and symptoms following severe acute respiratory process associated with an increased risk of venous thromboembolism, particularly in older individuals, men, and those with longer hospital stays and aggressive treatments (11). While the rate of post-discharge thrombotic events in COVID-19 patients is lower compared to those during hospitalization, these events may be more related to “immunothrombosis” following the recent infection rather than true thrombotic events (12, 13).

The pathophysiology of post-COVID is not yet fully defined, with several hypotheses proposed, including immune dysregulation, viral persistence, endothelial dysfunction, microthrombosis, and their consequences (14). Identified risk factors include gender, the number of symptoms during the acute phase, and comorbidities, while vaccination and Omicron infection are associated with a lower prevalence (6).

Neutrophil extracellular traps (NETs) are web-like structures composed of DNA, histones, and antimicrobial proteins, released by activated neutrophils to capture and eliminate pathogens. Recent studies have demonstrated that excessive NET formation, a process known as NETosis, can occur in COVID-19 patients, as in other viral infections, and may contribute to hypercoagulability and thrombus formation (15–17). Additionally, macrophages play a crucial role in the immune response to viral infections, displaying either pro-inflammatory or anti-inflammatory phenotypes depending on the stimulus.

To develop prognostic or diagnostic models and understand the underlying mechanisms of thrombotic events in COVID-19, it is crucial to assess the association between NETosis markers, macrophage activity, and disease outcomes (18–20). Additionally, comparing NETosis marker levels and macrophage activity across different waves of the pandemic can offer valuable insights into temporal changes in immune response and disease severity. Moreover, examining the response to infection, coagulation, and macrophage activity markers at various stages of infection may help identify potential biomarkers for disease progression and treatment response. The aim of this study is to explore the impact of NETs and macrophage activation in SARS-CoV-2-infected patients who developed complications.

## 2 Patients and methods

### 2.1 Study design

The study was conducted in collaboration with several hospitals, including the Translational Research Unit at the Research Health Institute of Aragón (IIS Aragón), which serves the general population. Hospital settings provide an ideal environment for this investigation, focusing on participants diagnosed with COVID-19. Our study included both male and female participants.

The study employed a comparative retrospective-prospective design, involving two distinct pandemic waves in Spain: the initial wave in March 2020 and the subsequent wave in July 2021 (21). A group of 100 healthy controls, matched for sex and age, was also included. These controls were selected from individuals prior to the pandemic infection. The longitudinal design included assessing responses to infection, hemostatic parameters and liver enzymes at two critical time points, at diagnosis and after seven days, to assess temporal variations in immune response patterns and their correlation with disease severity. In the first-year follow-up, we explored developing of thrombotic events and persistence of post-COVID syndrome.

The study was accepted by the Research Ethics Committee of the Community of Aragon (CEICA. 20/355) and it complied with the European General Data Protection Regulation (GDPR 2016/679) and LO 3/2018. 500 µL of plasma were obtained from whole blood collected in EDTA tubes from healthy controls and patients included in this study. Samples were stored at -80°C and were provided by the Biobank of the Aragon Health System, integrated in the Spanish National Biobanks Network (PT20/00112) and they were processed following standard operating procedures with the appropriate approval of the Ethics and Scientific Committees. The project has followed the ethical principles established in the Declaration of Helsinki - Ethical Principles for Medical Research Involving Human Subjects.

### 2.2 Data source

Data collection relied on comprehensive reviews of medical records, encompassing patient demographics, clinical presentations and laboratory test results.

### 2.3 Inclusion criteria

- Confirmed COVID-19 diagnosis through laboratory testing, upon arrival at the hospital in March 2020 and in July 2021.
- Age range between 18-75 years.
- Admission to a hospital or specialized medical center for COVID-19 treatment was warranted after individualized assessment in the emergency department, as the condition was classified as severe.
- Availability of comprehensive medical records and laboratory test results.

### 2.4 Exclusion criteria

- Patients with a history of pre-existing clotting disorders.
- Patients with a history of autoimmune diseases or previous immunosuppression.
- Pregnant or breast-feeding females.
- Patients with incomplete medical records or missing laboratory test results.
- Patients who were not hospitalized.

### 2.5 Demographic, clinical and laboratory data

Clinical data were retrospectively collected from medical records, including patient demographics, analytical data, radiological findings at diagnosis, and follow-up information. This included associated comorbidities, thrombotic events, SARS-CoV-2 vaccination status, mortality, and post-COVID-19 syndrome outcomes, defined as the persistence of clinical manifestations related to the infection. The general characteristics of the variables are detailed in **Supplementary Table S1**.

Basic analytical data were obtained at diagnosis and seven days after: blood counts (Beckman Coulter DxI 9000), hemostatic parameters (ACL-TOP 500. Werfen), including prothrombin time (PT), activated partial thromboplastin time (aPTT), fibrinogen and D-dimer; pro-BNP, pro-calcitonin, IL-6, ferritin, liver enzymes measuring alanine aminotransferase (ALT) and aspartate aminotransferase (AST), gamma-glutamyl transferase (GGT) and lactodehidrogenase (LDH) (Roche-Diagnostics cobas 6000 modular analyzer).

### 2.6 Biomarkers

Macrophage activation biomarkers included the determination of the chitotriosidase enzymatic activity (ChT) using the artificial substrate 4-methylumbelliferyl-β-DN, N´, N´´ triacetyl-chitotrioside (4 MU-chi-totrioside, Sigma Chemical Co, St Louis, MO, USA) according to the protocol described by Hollak et al, 1994. CCL18/PARC and YKL-40 were determined by Human CCL18/PARC DuoSet ELISA and Human Chitinase 3-like 1/YKL-40 DuoSet ELISA, respectively according the manufacturer’s instructions (Bio-Techne R&D Systems).

NETosis biomarkers included myeloperoxidase (MPO) (Human Myeloperoxidase DuoSet ELISA), neutrophil elastase (NE) (Human Neutrophil Elastase/ELA2 DuoSet ELISA), p-selectin (P-SEL) (Human P-Selectin/CD62P DuoSet ELISA) and S100A8/S100A9 heterodimer (MPR) (Human S100A8/S100A9 Heterodimer DuoSet ELISA) determination that was performed by immunoassays according to the manufacturer’s instructions (Bio-Techne R&D Systems). Circulating free DNA (cfDNA) and DNase were determined by fluorimetry with SYTOX™ Green Nucleic Acid Stain and DNaseAlert™ QC System, respectively according to the manufacturer’s instructions (Invitrogen™, Waltham, USA).

ELISA (Enzyme-Linked ImmunoSorbent Assay) immunoquantification assays were initiated by incubating the primary antibody, at the concentration specified by the manufacturer, in a clear 96-well plate (MaxiSorp F8x12 wells plate, ThermoFisher) in the dark, overnight. After overnight incubation, the plate was washed with the wash solution (900 mL distilled water, 100 mL 10x PBS (Lonza), and 500 µL of Tween 20 (Sigma-Aldrich)) and 300 µL per well of blocking solution (90 mL distilled water, 10 mL 10x PBS (Lonza), and 1 g BSA (Sigma-Aldrich)) was added. After one hour under agitation, the plate was washed again, and 100 µL of sample was added to each well, followed by incubation for 2 hours under agitation. After 2 hours and another washing step, the secondary antibody, at the concentration indicated by the manufacturer, was added and incubated under agitation for an additional 2 hours. After washing the plate, 100 µL of streptavidin was added to each well and incubated under agitation for 20 minutes. After this period, the plate was washed again with the wash solution, and 100 µL of TMB chromogenic solution (Zeulab) was added to each well, followed by 20 minutes of incubation under agitation in the dark. The final step was to add 50 µL of STOP solution (Zeulab) to each well to stop the reaction, incubating for 10 minutes again under agitation and in the dark. After 10 minutes, the absorbance of the plate was read using the Synergy HTX plate reader (Biotek) at a wavelength of 450 nm, with a reference reading at 550 nm.

Circulating free DNA (cfDNA) was quantified following the manufacturer’s instructions. A known concentration of DNA was used to generate the calibration curve, from which 100 µL of each point was added in duplicate. For the samples, 100 µL of sample, diluted 1:10 with 1x PBS (Cytiva), was added to a black 96-well plate (FluoroNunc MaxiSorp F96, ThermoFisher). Subsequently, 100 µL of SytoxGreen (ThermoFisher) at 4 mM was added to the calibration curve and samples. The blank for each sample was prepared by adding 100 µL of sample diluted 1:10 with 1x PBS (Cytiva) and 100 µL of 1x PBS (Cytiva) to the well instead of SytoxGreen (ThermoFisher). Finally, the plate was incubated at 25°C for 10 minutes in the Synergy HTX plate reader (Biotek), followed by fluorescence reading with an excitation wavelength of 485 nm and emission wavelength of 528 nm.

For DNase quantification, the necessary reagents were prepared according to the instructions of the commercial kit (ThermoFisher). The calibration curve was generated using DNase I enzyme. The substrate tube provided by the kit was reconstituted with 1 mL of 1x TE. The assay plate used was a black 96-well plate (FluoroNunc MaxiSorp F96, ThermoFisher). 10 µL of substrate was added to all wells, followed by the addition of 90 µL of the calibration standards and 80 µL of undiluted samples to the corresponding wells. Finally, 10 µL of 10x nuclease buffer was added to all wells, except for those containing the calibration standards. Once the plate preparation was completed, it was incubated in the Synergy HTX plate reader (Biotek) for 25 minutes at 37°C, followed by fluorescence reading with an excitation wavelength of 530 nm and an emission wavelength of 590 nm.

### 2.7 Statistics

Statistical analyses were performed with SPSS 21.0 software using non-parametric tests for independent and related samples. The sample size for this study was determined through a power analysis, considering the expected effect size and desired power level (0.05). After observing that the obtained data did not follow a normal distribution, the non-parametric statistical test U of Mann-Whitney was used to analyze whether there were statistically significant differences between COVID-19 patients and healthy controls. This test was also used to study possible differences between patients who had experienced complications and those who had not. Statistical Wilcoxon test for paired samples was used to analyze differences between samples from the same wave. Finally, to study the correlation between the different biomarkers, the Pearson correlation test was used.

## 3 Results

### 3.1 Patients included

A cohort of 60 patients was enrolled in the study, with 30 patients enrolled in March 2020 and the other 30 in July 2021. Additionally, 100 healthy controls samples obtained from Aragon Biobank before May 2019, matched by gender and age, were included in the analysis. The Laboratory normal ranges from healthy controls of analytical data, macrophages activation and NETs formation biomarkers are detailed in **Supplementary Table S4**.

The median age of the patients in the first wave was 71.0 years (Q1-Q3: 53.0-89.0), while in the second wave it was 41.5 years (Q1-Q3: 34.0-50.00). The gender distribution in the first cohort was 47% females and 53% males, and in the second cohort it was 60% females and 40% males; detailed data can be found in **Supplementary Table S1**.

Two plasma samples from each of the 60 patients with SARS-CoV-2 infection were provided by the Aragon Health System Biobank (30 for each wave), with the first sample collected just after diagnosis and the second one collected seven days later. The follow-up has been carried out over one year from diagnosis for each patient, with the objective of determining the development of short and medium-term complications, such as death, thrombosis and symptoms related to post-COVID-19 syndrome.

### 3.2 Clinical data

**Figure 1:**
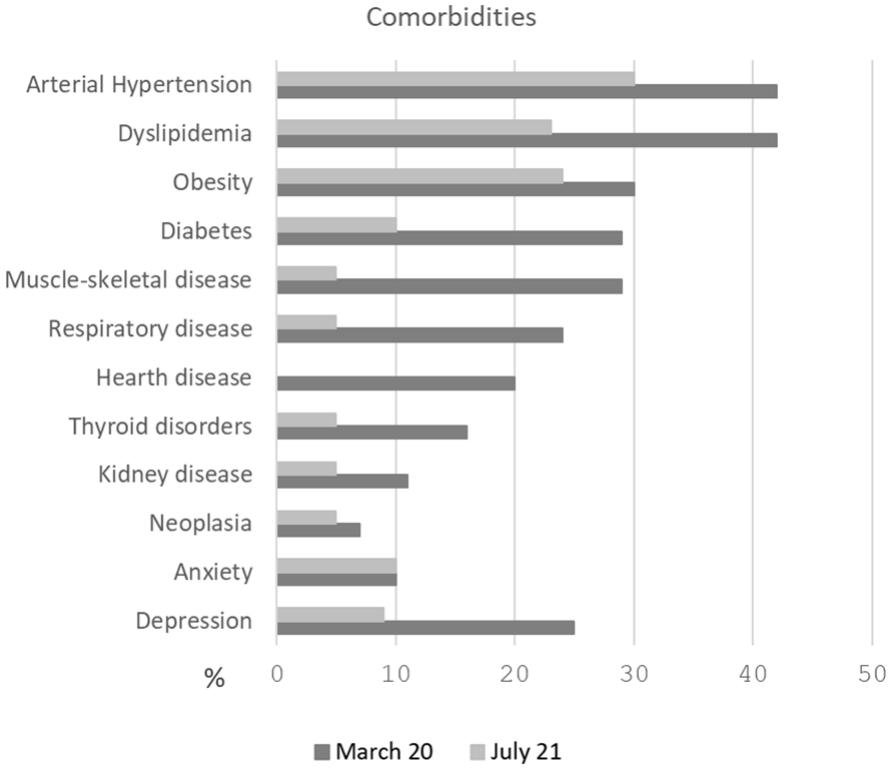
Percentage of comorbidities in both cohorts. The first cohort (March 2020) showed a higher prevalence of pre-existing conditions, with the most common being arterial hypertension and dyslipidemia.

All patients were admitted to the hospital with fever and respiratory symptoms. The first cohort showed a higher prevalence of pre-existing conditions and comorbidities, with the most common being arterial hypertension and dyslipidemia. The percentage of comorbidities in both cohorts is shown in **Figure 1**.

**Figure 2:**
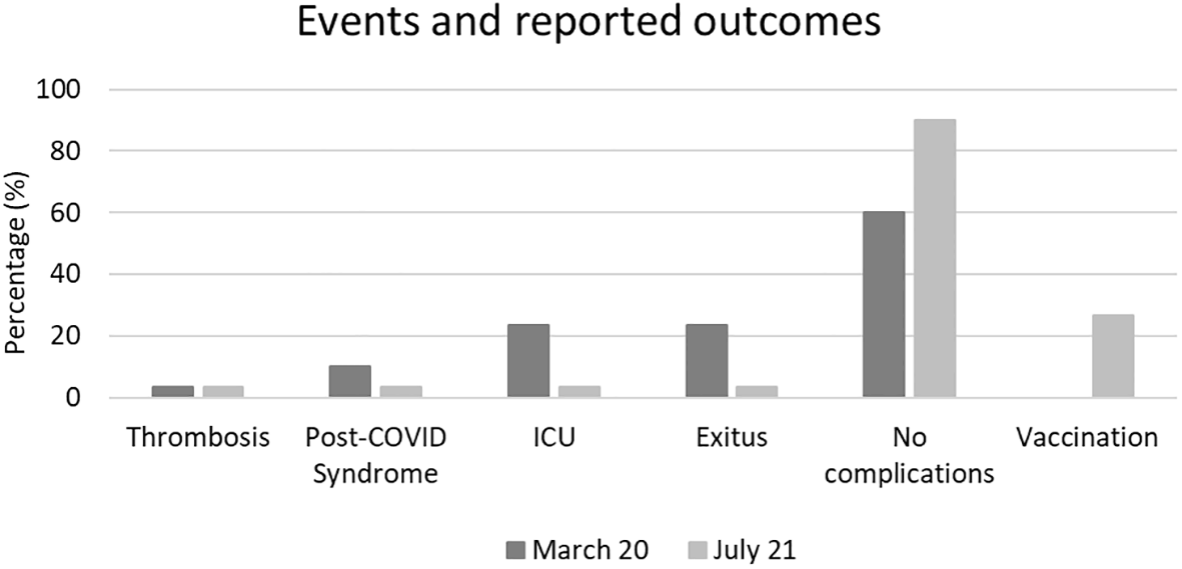
Incidence of events and reported outcomes during follow-up. A higher incidence of post-COVID syndrome, ICU admissions, and mortality was observed in patients from first wave compared to the second.

Additionally, the incidence of post-COVID syndrome was higher during the first wave (10% vs. 3%), as were the rates of ICU admission (27% vs. 3%) and mortality (27% vs. 3%). In the second wave, only 26.6% of patients (8 individuals) had received prior vaccination. During follow-up, after July 2021 a total of 42 patients from both waves were vaccinated. The incidence of events and reported outcomes during follow-up are detailed in **Figure 2**.

### 3.3 Analytical tests

**Figure 3:**
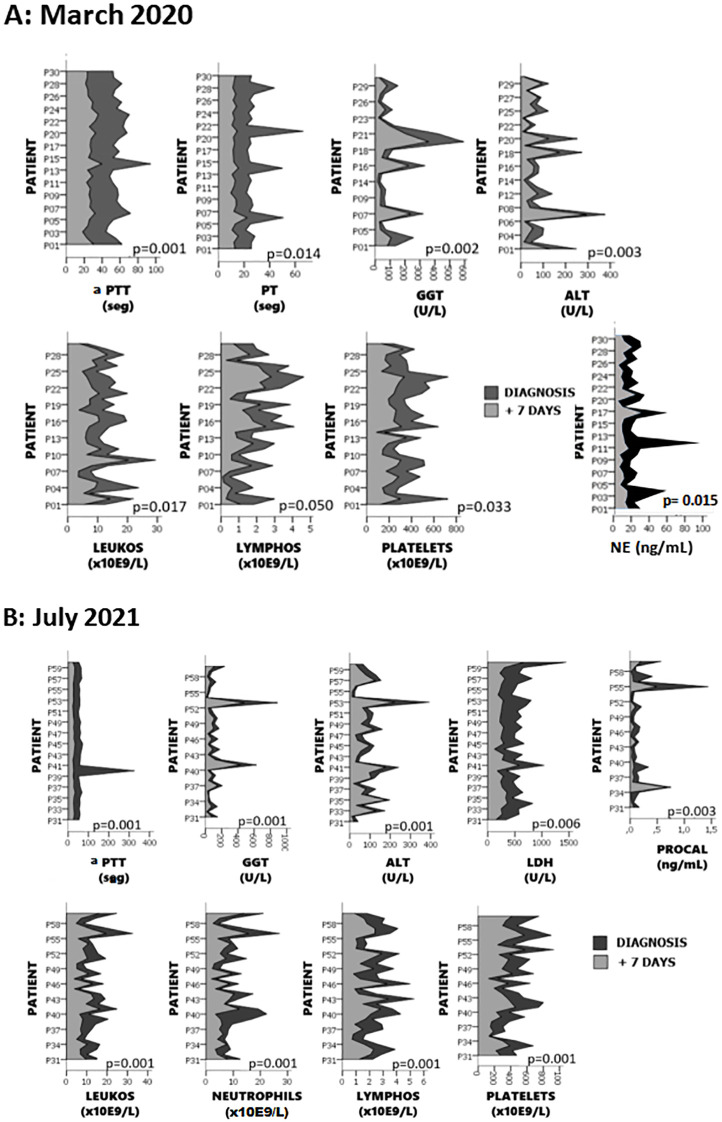
Differences in analytical parameters observed 7 days after diagnosis. Panel **(A)** shows parameters with significant changes in the first wave, and panel **(B)** presents those in the second wave. Significant differences were found in leukocytes, lymphocytes, platelets, aPTT, PT, ALT, GGT and NE in the first wave, and in leukocytes, neutrophils, lymphocytes, platelets, aPTT, ALT, GGT, LDH, and pro-calcitonin (PRO-CAL) in the second wave.

Detailed basic analytical measurements performed in both cohorts, at diagnosis and seven days later, are provided in **Supplementary Table S2**. In **Figure 3**, only the parameters that showed significant differences seven days post-diagnosis are presented. In the first wave, we observed differences in leukocytes, lymphocytes, platelets, aPTT, PT, ALT, GGT.and neutrophil elastase (NE) In the second wave, significant differences were found in leukocytes, neutrophils, lymphocytes, platelets, aPTT, ALT, GGT, LDH, and pro-calcitonin (PRO-CAL) (**Figures 3A, B**). When comparing between the two waves, the only significant difference was observed in fibrinogen, which showed a notable increase in the second wave compared to the first (p=0.0040).

### 3.4 Biomarkers

**Figure 4:**
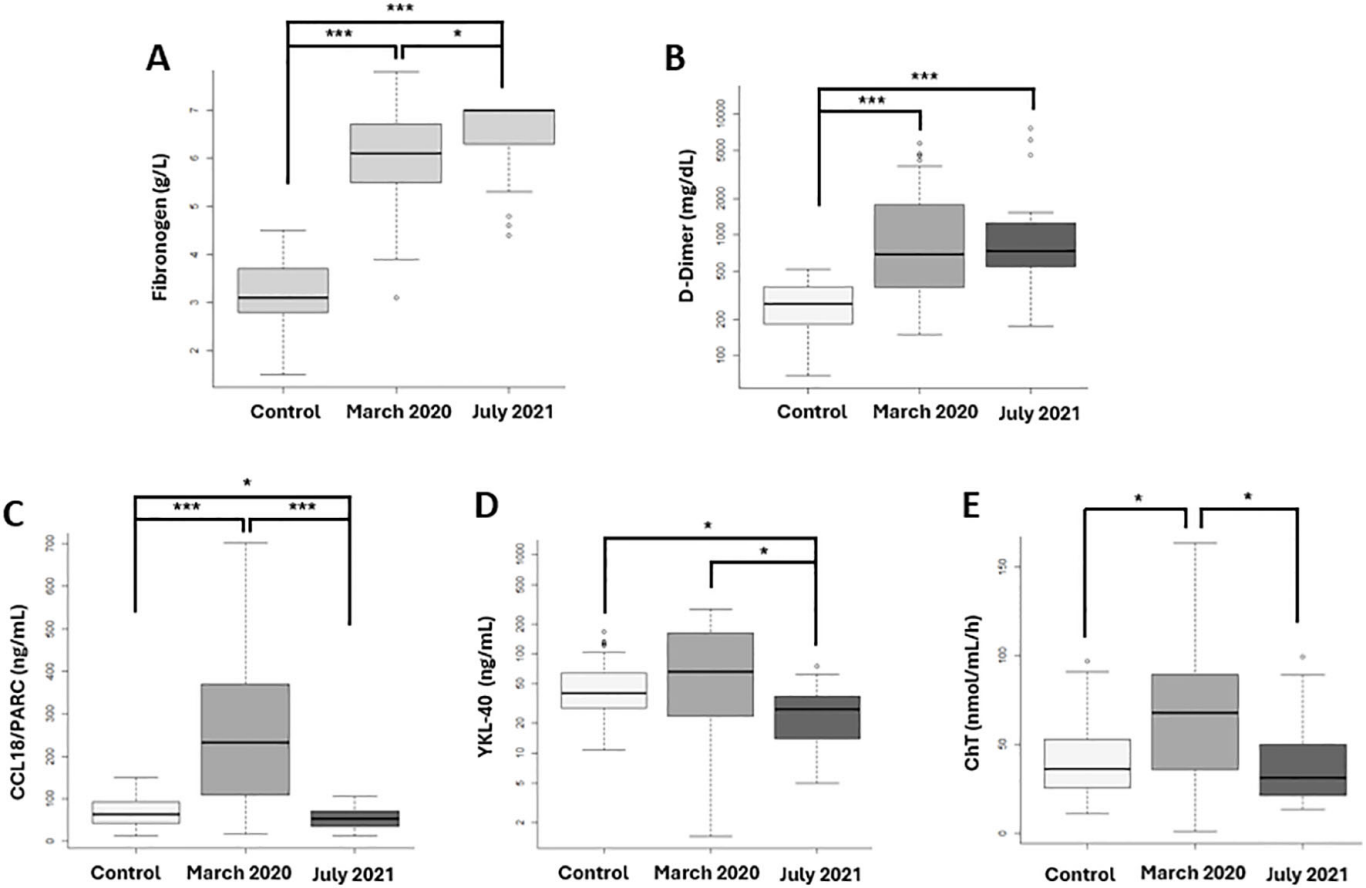
Fibrinogen, D-Dimer and Inflammation biomarkers related macrophage activation at SARS-CoV-2 diagnosis compared with 100 prepandemic controls. First vawe patient: N= 30 and second vawe patients: N=30). **(A)** Fibrinogen and **(B)** D-Dimer, proteins related to the hemostatic process, show significant increases in both waves compared to controls, with fibrinogen being significantly more elevated in the second wave. Macrophage activation biomarkers: **(C)** CCL18/PARC, **(D)** YKL-40, and **(E)** Chitotriosidase enzyme (ChT) demonstrate significantly higher levels in the first wave compared to controls and the second wave. *p-value <0.05; *** p-value <0.0001.

Regarding inflammation and macrophage activation biomarkers at diagnosis, a significant increase in fibrinogen was observed in both the first and second wave compared to controls (p=1x10^-9^; p=1x10^-11^, respectively), as well as between the two waves (p=0.0038) (**Figure 4A**). Concerning D-Dimer, significant increases were found in both the first wave (p<0.0001) and second wave (p<0.0001) compared to controls, but no between the two waves (**Figure 4B**). For the cytokine CCL18/PARC, significant increases were found when comparing the first wave to controls (p<0.0001) and to the second wave (p<0.0001). Additionally, a significant decrease in CCL18/PARC was observed in the second wave compared to controls (p=0.0379) (**Figure 4C**). A significant decrease in YKL-40 was detected in the second wave compared to the first wave (p=0.0281) and to controls (p=0.0014) (**Figure 4D**). Finally, a statistically significant increase in ChT was observed in first-wave patients compared to controls (p=0.0047) and to second-wave patients (p=0.0032) (**Figure 4E**).

Detailed raw data for each marker at diagnosis and after seven days in each wave are provided in **Supplementary Table S3**.

Median and quartiles of NETs formation biomarker data are presented in **Table 1**. The normal ranges for all biomarkers are detailed in **Supplementary Table S4**.

**Table 1:** Neutrophils extracellular traps (NETs) biomarkers in patients at diagnosis and controls represented as median (Q1-Q3).

Biomarkers	Control	1^st^ wave patients	2^nd^ wave patients
**MPO (ng/mL)**	42.2 (28.7 – 58.6)	177.0 (106.3 – 323.5)	82.4 (14.9 – 262.4)
**NE (ng/mL)**	6.1 (4.4 – 8.0)	20.5 (17.0 – 29.6)	49.8 (18.4 – 76.8)
**P-SEL (ng/mL)**	28.4 (23.6 - 35.9)	22.5 (14.2 – 27.3)	13.9 (8.6 – 16.2)
**MRP (ng/mL)**	111.2 (51.4 – 176.4)	474.5 (215.6 – 1023.6)	327.7 (138.5 – 808.5)
**cfDNA (ng/mL)**	0.4 (0.3 – 0.6)	0.3 (0.3 – 0.4)	0.4 (0.3 – 0.5)
**DNAse (U/L)**	1000.9 (849.3 – 1145.0)	1442.6 (1321.6 – 1599.6)	2573.2 (2464.0 – 2642.6)

**Figure 5:**
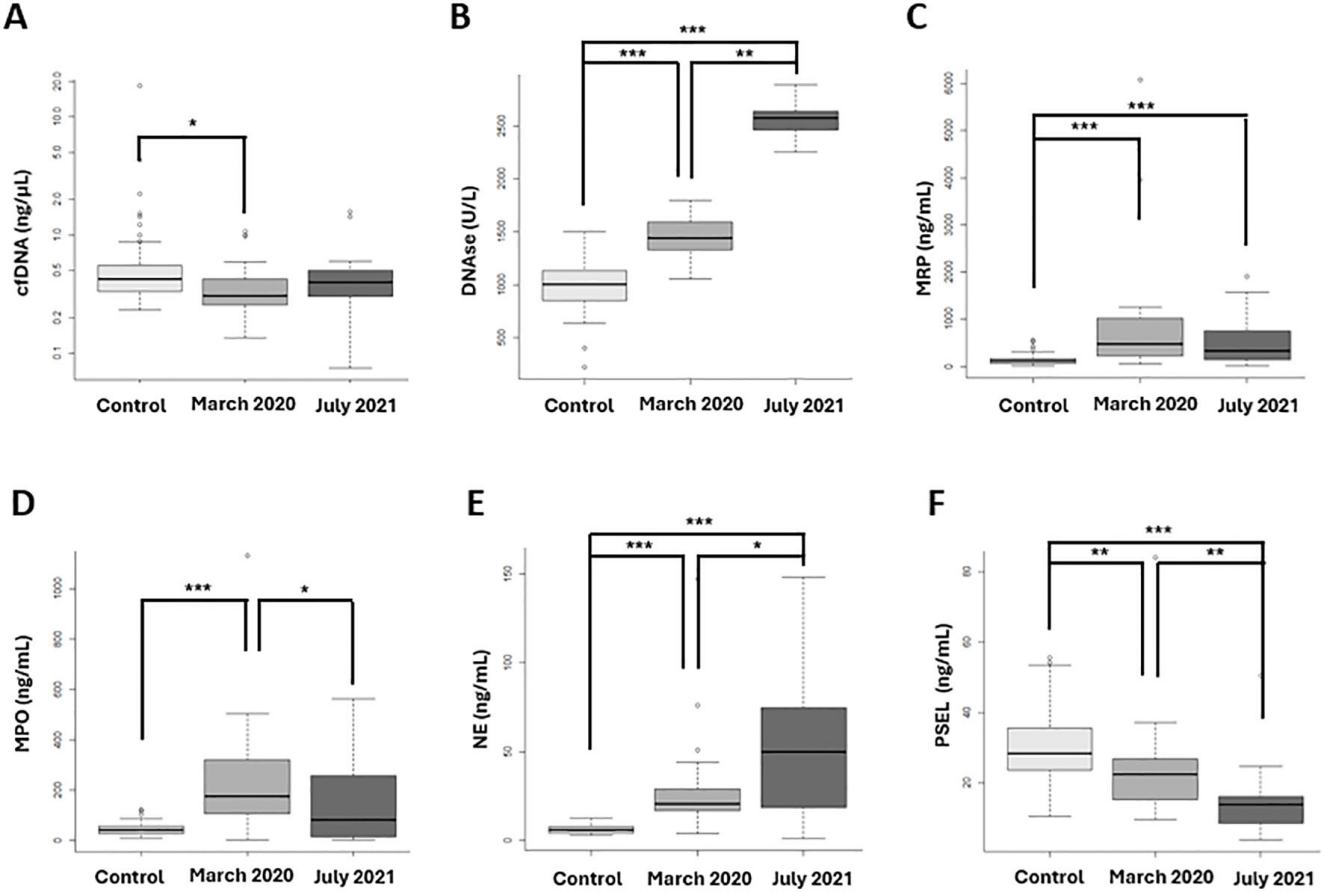
NETosis biomarkers at diagnosis, between the two vawes and healthy controls. **(A)** cfDNA levels significantly decreased in the first wave compared to controls. **(B)** DNase concentrations were significantly lower in the first wave compared to the second wave and controls. **(C)** MRP concentrations significantly increased in both the first and second waves compared to controls, with no significant difference between the two waves. **(D)** MPO levels significantly increased in the first wave compared to controls and the second wave. **(E)** NE levels were significantly lower in the first wave compared to the second wave. **(F)** P-SEL concentrations were significantly lower in the second vawe. **(A)** cfDNA; **(B)** DNAse; **(C)** S100A8/S100A9 heterodimer (MPR); **(D)** Mieloperoxidase (MPO); **(E)** Neutrophil Elastase (NE); **(F)** P-selectine (PSEL). *p-value <0.05; **p-value <0.001; *** p-value <0.0001.

The **Figure 5** illustrates the differences observed in NETs biomarkers between groups. Significant findings include the following: A significant decrease in cfDNA levels was observed in the first wave compared to controls. (**Figure 5A**: cfDNA levels). Both waves showed increased DNase levels compared to controls, patients from the first wave exhibited significantly lower DNase concentrations compared to those from the second wave. (**Figure 5B**: DNase levels). MRP levels significantly increased in both the first and second waves compared to controls, with no significant difference between the two waves. (**Figure 5C**: MRP concentrations). A significant increase in MPO concentrations was observed in the first wave compared to both controls and the second wave (**Figure 5D**: MPO levels). Both waves showed increased NE concentration compared to controls and was significantly lower in the first wave compared to the second wave (**Figure 5E**: NE levels). Both waves showed decreased P-SEL levels compared to controls and were lower in the second wave. (**Figure 5F**: P-SEL levels).

In summary, when comparing patients between waves, significant differences were observed in NE, MPO, P-SEL, and DNase levels. Infected patients exhibited significant differences in NE, P-SEL, MRP, and DNase concentrations at diagnosis when compared to controls and between the two waves.

**Figure 6:**
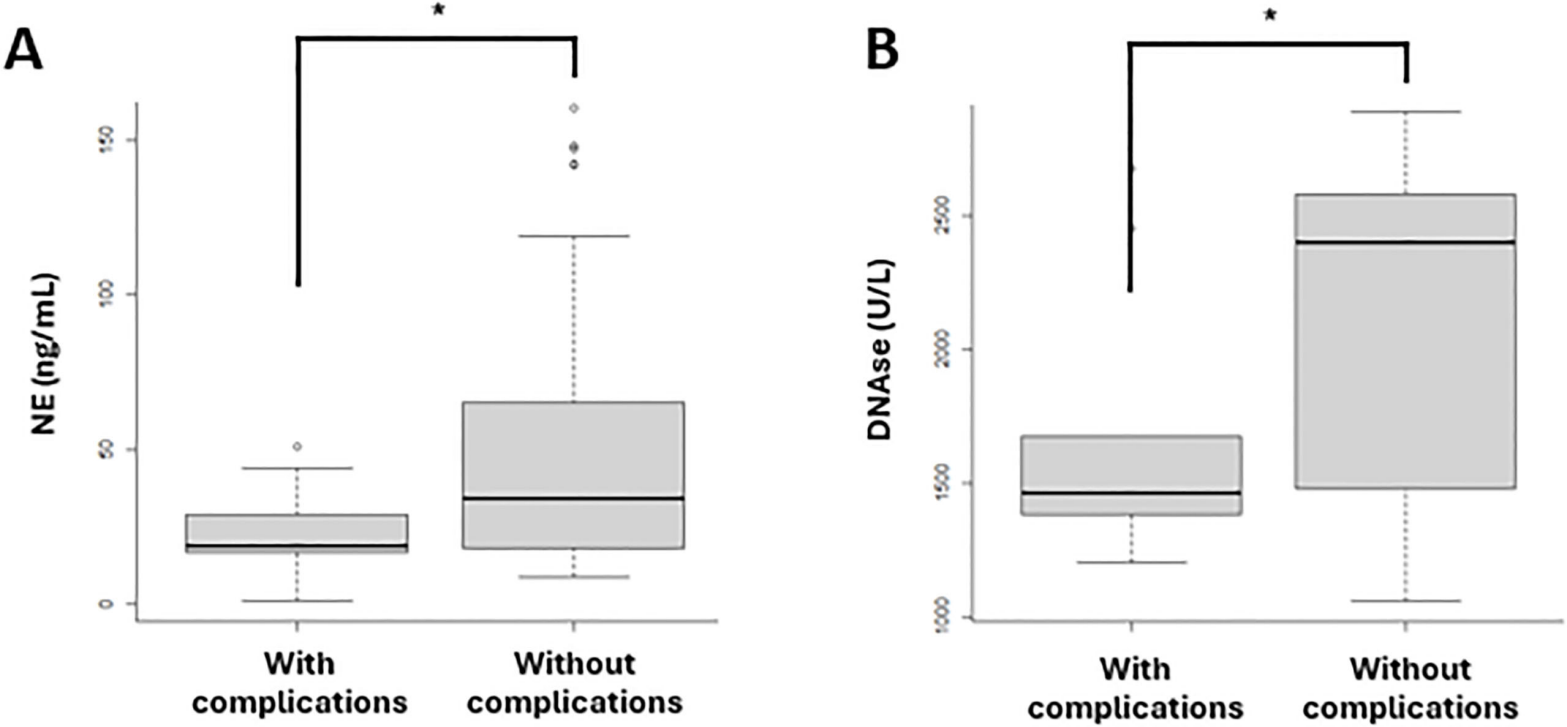
Biomarkers of NET formation in patients with (N=13) and without (N=47) complications in both vawes. **(A)** NE levels were significantly lower in patients who developed complications compared to those who did not. **(B)** DNase concentrations were significantly decreased in patients with complications compared to those without complications. The complications included thrombosis, post-COVID syndrome, ICU admission and mortality. **(A)** Neutrophil elastase (NE); **(B)** DNAse. *p-value <0.05.

The biomarkers related to macrophage activation and NET formation, as mentioned above, were analyzed in samples collected at diagnosis from patients in both waves. These biomarkers were compared between patients who developed complications (N=13) during follow-up and those who did not (N=47). The complications included thrombosis, post-COVID syndrome, ICU admission, and mortality. In patients who developed complications, a significant decrease in NE (p=0.0470) (**Figure 6A**) and DNase (p=0.0313) (**Figure 6B**) was observed compared to those who did not develop complications.

The analysis of the profile of macrophage activation and NETs formation biomarkers in vaccinated patients included in the second wave (N=8) compared to unvaccinated (N=22) at the time of diagnosis in these patients, we have observed an increase in the concentration of CCL18/PARC biomarker in vaccinated (p=0.001) compared to unvaccinated patients, although the median values ​​in both groups are within the normal range. **Supplementary Figure S1A**. When comparing vaccinated patients (N=8) with the total unvaccinated patients (N=52) from both waves, a significant increase in DNase (p=0.009) and YKL-40 (p=0.039) was observed in vaccinated patients. In contrast, unvaccinated patients showed a significant indecrease in P-SEL concentration(p=0.023), compared to vaccinated patients. **Supplementary Figure S1B**.

### 3.5 Correlation between biomarkers

The correlation between biomarkers and various analytical variables is shown in **Table 2**. A significant correlation was observed between P-SEL and blood cell counts. Additionally, significant correlations were found between NETosis biomarkers (MRP and cfDNA) and several analytical variables, including D-dimer, liver enzymes, LDH, and ferritin.

**Table 2:** Correlation between variables.

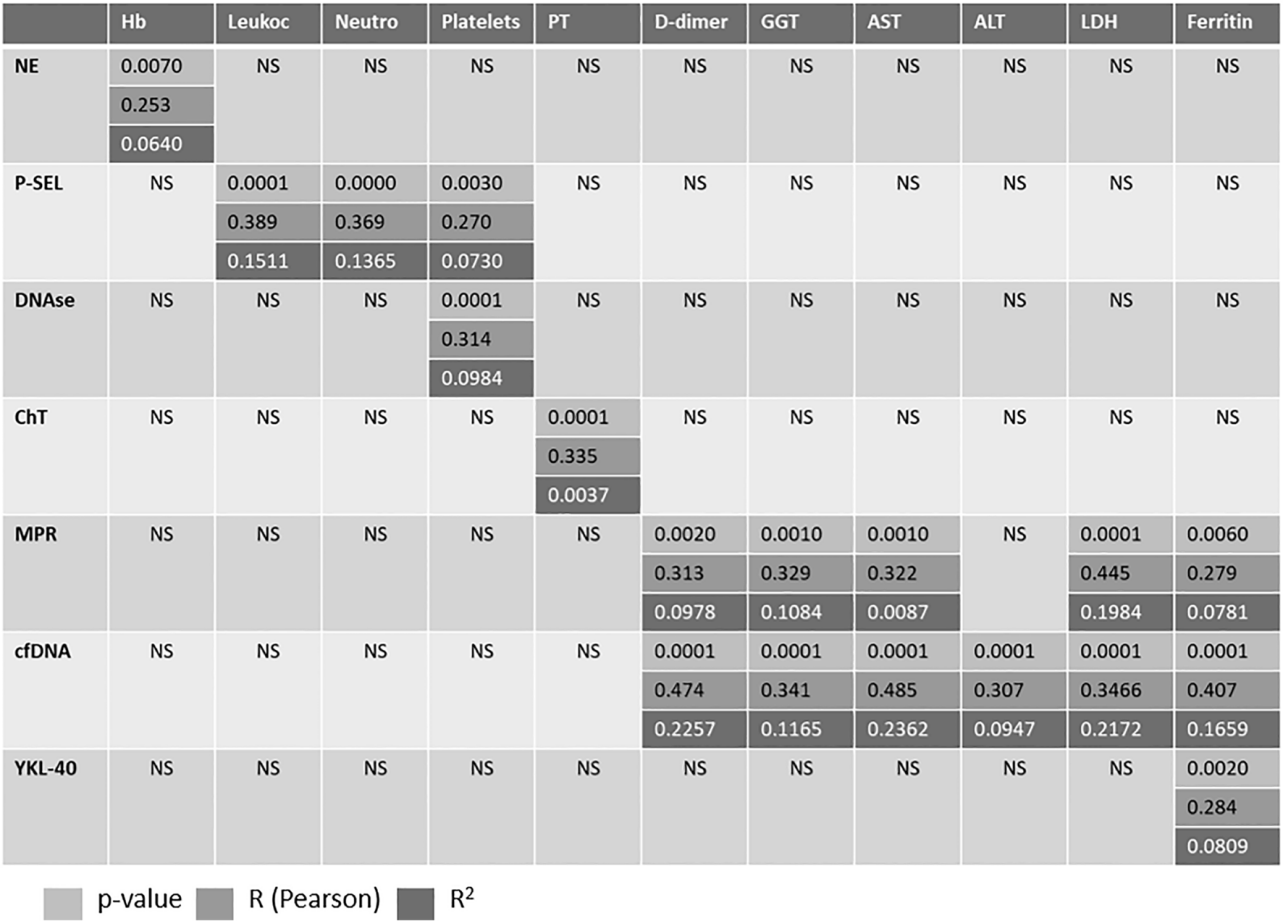

## 4 Discussion

The global incidence of COVID-19 pandemia has resulted in over 5.57 million deaths worldwide (22). The virus has evolved through variants like alpha, beta, gamma, delta and the highly transmissible Omicron. Regarding the SARS-CoV-2 virus lineages, the dominant variant in Spain during the first wave was BA.1 (Alpha), as it was in other European countries. Starting in January 2021, the BA.2 (Delta) variant began circulating, reaching its peak in August 2021. By July 2021, however, the Alpha variant (B.1.1.7) was still predominant (21). The BA.2 variant was considered to have had a greater impact due to its increased transmissibility, potential for more severe infections, and/or higher lethality. This resulted in a decreased effectiveness of the natural immune response, a reduction in the diagnostic capacity of available tests, and diminished effectiveness of treatments. Despite this, Spain have presented a paradox: the population’s immunity was considered very high due to the extensive exposure since the start of the pandemic, the high vaccination coverage (over 90% of people aged 60 or older), and the vaccine’s effectiveness in preventing hospitalization and death, which was close to 90%.

At present, the diffusion of vaccination and the exposure of the population to the virus has weakened the aggressiveness of the infection and greatly reduced the incidence of complications. Nevertheless, the pathophysiology of COVID-19 continues to serve as a model for understanding airborne viral infections. SARS-CoV-2 can activate both innate and adaptive immune responses, potentially triggering an exaggerated cytokine storm that leads to septic shock, acute respiratory distress syndrome, and/or multiple organ failure in critically ill patients (23–25). A large body of literature has been published on the impact of this viral infection on the immune system’s behavior and activity, including several systematic reviews (26, 27). The published studies mainly focus on the excessive inflammatory response triggered by viral infection, and the effects on lymphocyte populations and cytokine release. However, the mechanisms underlying this dysregulated immune response remain poorly understood, there is limited information on the macrophage response to this infection and the changes in this kind of response in vaccinated individuals. It is evident that the reaction of macrophages and neutrophils to infection is exaggerated leading to excessive cytokine production and neutrophil activation. This, in turn, contributes to the most severe complications of the infection, including the formation of multiple microthrombi and endothelial damage (5, 9). In our study, these complications were particularly evident during the first wave.

These factors can significantly increase morbidity and mortality rates in COVID-19 patients, particularly those with preexisting cardiovascular conditions. A review of the increased levels of NETs in the bloodstream of patients with severe COVID-19 suggests that NETs may play a significant role in the disease’s progression and severity, contributing to inflammatory and thrombotic complications (27–30). Despite the components of NETs being nonspecific and potentially causing tissue injury, either directly or by increasing the pro−inflammatory response, there have been numerous discussions about the hyperinflammatory situation caused by infection especially in the early phase of the pandemic. This includes arguments regarding the overproduction of antibodies and the cytokine storm leading to severe organ damage and increased risk of thrombosis or microthrombosis (28, 29).

In the review study by Behzadifard M et al. (30), which analyzed 55 articles on neutrophil extracellular traps (NETs), it was highlighted that NETs play a pivotal role in host defense against pathogens (30). However, their overproduction can result in the failure of self-tolerance and activation of the immune system, contributing to the development of autoimmunity. Autoimmune phenomena have been observed in many COVID-19 patients, with a pathophysiology like that of other viral infections that induce NET formation. The authors conclude that modulating the formation and degradation of NETs could potentially reduce the severity of the disease. Another study conducted during the first wave in our region as our research shows a relationship between certain NET markers and the severity of the infection, although it examined different parameters than those included in our study (31).

Our retrospective-prospective comparative study focuses on the role of neutrophil in investigating potential changes in these immune mechanisms over time. We aim to explore the impact of neutrophil extracellular traps and macrophage activation on patients infected by SARS-CoV-2 who had developed a complication. Using a comparative design, we analyze patients from the initial wave in March 2020 and the subsequent wave in July 2021 in our region. This approach allows the evaluation of temporal changes in immune responses and disease severity, considering the application of early care protocols and the dissemination of vaccination (32). It is important to mention that the first vaccination had already taken place by the time of the second wave, and the episodes during this period appeared to be less aggressive. However, in this second cohort, only 8 patients had been vaccinated. There are a lower rate of complications and a lower increase in both inflammatory and some NETosis-related biomarkers (MRP, MPO, P-SEL). In relation to the episodes of thrombosis, they were similar in both groups and occurred at a low rate (3%), possibly justified by the early administration of low molecular weight heparin at the time of diagnosis, according to the recommendations of scientific societies (33).

It must be assumed that the profile of patients infected in the first wave was different from the second wave; they were significantly older (71 years vs 41.5 years, p<0.001) and presented higher incidence of comorbidities. Both factors undoubtedly contributed to a longer length of hospital stay, a higher number of patients requiring ICU care (27% vs 3%), and a greater number of mortality (27% vs 3%). The incidence of post COVID syndrome was also higher in the first wave (10% vs 3%). Post-COVID syndrome increases venous thrombosis risk post-discharge, especially in older men with longer hospital stays, aggressive treatment, lack of thromboprophylaxis, and persistent prothrombotic state; this situation warrants close monitoring and extended thromboembolic prophylaxis (32–35).

COVID-19 patients underwent a basic laboratory analysis at the time of infection diagnosis and again seven days later. In both waves, patients exhibited significantly elevated levels of blood cells, liver enzymes, and coagulation markers (PT and PTT) at the time of diagnosis compared to the samples taken seven days later, observing the improvement of patients both clinically and analytically.

The inclusion of macrophage and NETosis biomarkers enhances the depth of analysis, providing valuable insights into the immune responses associated with thrombotic events. The standardized approach to data collection ensures the reliability and consistency necessary for robust data analysis and interpretation. Concerning the evaluation of biomarkers related to inflammation process, we have observed an increase in potent biomarkers of macrophage activation as the activity of chitotriosidase enzyme and the cytokine CCL18/PARC (Pulmonary and activation-regulated chemokine also named CC-chemokine ligand 18). These biomarkers have been shown to increase in inflammatory processes of the respiratory tree (36). The same is true for other inflammatory conditions or lysosomal storage diseases, where macrophages are the target cells, such as Gaucher disease, acid sphingomyelinase deficiency or others (37). In this study, both biomarkers were increased among controls and patients across both waves. A significant difference was found between the two waves, showed higher levels in the first wave, supporting the inflammatory state underlying the process. Nevertheless, during the first wave, a greater aggressiveness of the process was inferred.

YKL-40 is a chitinase-like protein with demonstrated increment in the lung and circulation of patients with severe asthma (38). In our study, significantly reduced values of this molecule were observed in patients from the second wave compared to those from the first wave and the control group. These results may indicate a lower degree of pulmonary involvement and a reduced inflammatory component in patients from the second wave, which aligns with the data showing fewer cases of complications during the second wave.

Another protein also involved in inflammatory processes, D-Dimer, that is universally determined in emergency departments, showed a significant increase between controls and patients in each wave but not correlated with macrophage biomarkers analyzed. D-Dimer is a protein that is increased in other situations such as severe infections, neoplasms, liver disease, ageing and is not useful in the diagnosis of coronavirus infection but is helpful for predicting thromboembolic events (4, 39). However, in this study the NETosis markers heterodimer and circulating free DNA do correlate with D-Dimer, which may indicate a greater influence of the release of intracellular elements on the risk of microthrombosis than reliance on cytokine stimulation. Fibrinogen determination is also routinely performed in the protocols of patients who come to the emergency department. In this cohort it appears significantly increased in both waves and between the first and second wave. However, both D-Dimer and Fibrinogen tests in the Cochran review study have shown a very low level of evidence related to hypercoagulability in patients with severe SARS-CoV-2 infection (40). In this study, fibrinogen an acute phase reactant appears significantly elevated in the second wave compared to the first, but shows no correlation with the remaining variables. We consider it informative but a non-specific marker with a low degree of evidence in relation to the severity of the infection.

Since the production of microthrombi and endothelial damage are known complications of SARS-CoV-2 infection (41, 42), we evaluated several molecules associated with the NETosis process in patients infected during the first wave and compared them to those in the general population (pre-COVID) and in patients during the July 2021 wave. Our findings revealed statistically significant differences in several selected NETosis biomarkers when comparing COVID-19 patients to healthy controls. Specifically, we observed increased levels of NE, MPO, DNase, and MRP in the patients compared to controls. The role of these molecules—NE, MPO, and MRP—in the formation of NETs has been well established, and they are considered key biomarkers for these structures. In fact, studies have shown that inhibiting these molecules also prevents the development of NETs formation (43, 44). These results suggest an increased formation of NETs in COVID-19 patients. Furthermore, elevated concentrations of MPO and MRP have been linked to a higher risk of cardiovascular diseases, such as thrombosis (45–47).

The study by Jiménez-Alcázar et al. (48) demonstrated a relationship between NET formation and increased DNase levels, particularly in patients with thrombosis. Our findings align with this study, as we observed elevated DNase levels in COVID-19 patients compared to healthy controls. DNases are enzymes that hydrolyze the DNA within NETs, and thus, the increase in DNase levels could explain the decreased cfDNA levels observed in patients from the first wave. This observation coincides with the fact that elevated DNase levels observed in our study may reflect the body’s attempt to eliminate NETs and maintain cellular homeostasis, potentially to prevent vascular occlusion (49, 50).

The last significantly altered biomarker of NETs is P-SEL, which is found to be decreased in patients from both waves compared to controls and it decrease in the vaccinated patients is also striking. P-SEL is an adhesion molecule, which favors the interaction of white blood cells with the vascular endothelium. It has been shown to activate platelets, which are involved in the generation of NETs (49). Considering this information, an increase in P-SEL would be expected in our study. However, our results do not align with previously published findings. This discrepancy could be attributed to the administration of low molecular weight heparin at the time of diagnosis, as P-selectin has been reported to be inhibited by this therapy (51).

The term “long COVID” or “post-COVID syndrome” has various definitions, but it can generally be understood as the persistence of symptoms following infection, influenced by factors such as age, sex, disease severity, and follow-up time (44). Post-COVID syndrome is complex and has a notable association with thrombosis. Patients with this condition often exhibit dysregulated platelet responses and coagulation abnormalities, potentially due to the presence of a circulating plasma-derived molecule promoting thrombosis. However, a protective response seems to counterbalance this thrombotic tendency (45). Additionally, individuals recovering from severe COVID-19 are at higher risk of venous thromboembolism after discharge, as well as complications such as myocarditis, pericarditis, heart failure, arrhythmias (9, 33, 39), and thrombosis in various vascular territories, including the portal system.

Given the severity of the complications associated with SARS-CoV-2 infection, we examined whether macrophage activation biomarkers and NET formation were related to the development of these complications. When analyzing patients who developed thrombosis, post-COVID syndrome, were admitted to the ICU or died, we observed that they had lower levels of NE and DNase compared to those who did not experience complications. The decrease in DNase aligns with expectations, as this enzyme plays a crucial role in preventing vascular occlusions and maintaining cellular homeostasis (47, 48), so its decline could exacerbate the patient’s condition. In the case of NE, we would expect an increase, as previous studies have shown that NE activity is associated with exacerbations and a decline in lung function (51). The combination of decreased DNase and neutrophil elastase in COVID-19 patients suggests an imbalance in the regulation of the inflammatory response and neutrophil extracellular traps (NETs), which could complicate both inflammation and tissue repair. However, when DNase levels are downregulated, NETs are not adequately cleared, potentially promoting thromboinflammation and multi-organ damage. At the same time, neutrophil elastase, which contributes to both NET formation and tissue damage, also decreases. This may indicate a later stage of the disease in which neutrophils’ ability to combat infection is diminished, along with their microbicidal and tissue remodeling functions (52). In our study, we observed a significant decrease in DNase and neutrophil elastase levels at diagnosis in patients who later developed complications, these markers could therefore be considered risk factors for infection severity and linked to complications. Similar findings were reported by García et al. (52), who demonstrated that reduced functional DNases are risk factors for severe COVID-19, regardless of age, gender, or BMI, which are the major determinants of severe disease.

In summary, the concurrent decline in DNase and neutrophil elastase levels in our study reflects a depletion of the neutrophilic immune response and an inability to adequately regulate inflammation in patients infected and complications. This dysregulation may lead to a worse prognosis due to the persistence of NETs and their associated tissue damage.

## 5 Conclusions

In conclusion, our study reveals the complex relationship between neutrophil extracellular traps (NETs), macrophage activation, and the severity of COVID-19 complications. The immune response to SARS-CoV-2 infection evolves over time, especially between the first and second waves of the pandemic. During the initial wave, patients showed a more aggressive inflammatory response with elevated NETs and biomarkers, correlating with higher morbidity and mortality rates. In contrast, the second wave, marked by increased vaccination rates, demonstrated a significant reduction in inflammatory markers and complications, indicating that vaccination and improved treatments may have decreased disease severity.

Nevertheless, the study highlights the importance of monitoring NETs and macrophage activation as potential indicators of thrombotic events and post-COVID syndrome. The decline in DNase and neutrophil elastase levels in patients with severe complications suggests a dysregulated immune response that can worsen tissue damage and inflammation. This emphasizes the need for targeted therapies to modulate NET formation and degradation, potentially improving patient outcomes.

## Data Availability

The original contributions presented in the study are included in the article/**Supplementary Material**, further inquiries can be directed to the corresponding author/s.

## References

[1] Gupta A Marzook H Ahmad F. Comorbidities and clinical complications associated with SARS-CoV-2 infection: an overview. Clin Exp Med. (2023) 23:313–31. doi: 10.1007/s10238-022-00821- PMC8972750 35362771

[2] Camelo ALM Zamora Obando HR Rocha I Dias AC Mesquita AS Simionato AVC. COVID-19 and comorbidities: what has been unveiled by metabolomics? Metabolites. (2024) 14:195. doi: 10.3390/metabo14040195 38668323 PMC11051775

[3] Gardinassi LG Servian CDP Lima GDS Dos Anjos DCC Gomes Junior AR Guilarde AO. Integrated Metabolic and Inflammatory Signatures Associated with Severity of, Fatality of, and Recovery from COVID-19. Microbiol Spectr. (2023) 11:e0219422. doi: 10.1128/spectrum.02194-22 36852984 PMC10100880

[4] Al-Gburi S Beissert S Günther C. Molecular mechanisms of vasculopathy and coagulopathy in COVID-19. Biol Chem. (2021) 402:1505–18. doi: 10.1515/hsz-2021-0245 34657406

[5] Khan R Kuenzig ME Tang F Im JHB Widdifield J McCurdy JD. Venous thromboembolism after COVID-19 infection among people with and without immune-mediated inflammatory diseases. JAMA Netw Open. (2023) 6:e2337020. doi: 10.1001/jamanetworkopen.2023.37020 37812417 PMC10562941

[6] van der Made CI Simons A Schuurs-Hoeijmakers J van den Heuvel G Mantere T Kersten S. Presence of genetic variants among young men with severe COVID-19. JAMA. (2020) 324(7):663–73. doi: 10.1001/jama.2020.13719 32706371 PMC7382021

[7] Moschonas IC Tselepis AD. SARS-CoV-2 infection and thrombotic complications: a narrative review. J Thromb Thrombolysis. (2021) 52:111–23. doi: 10.1007/s11239-020-02374-3 PMC7810105 33449290

[8] Ackermann M Verleden SE Kuehnel M Haverich A Welte T Laenger F. Pulmonary vascular endothelialitis, thrombosis, and angiogenesis in covid-19. N Engl J Med. (2020) 383:120–8. doi: 10.1056/NEJMoa2015432 PMC7412750 32437596

[9] Ali MAM Spinler SA. COVID-19 and thrombosis: From bench to bedside. Trends Cardiovasc Med. (2021) 31:143–60. doi: 10.1016/j.tcm.2020.12.004 PMC7836332 33338635

[10] Tomar B Anders HJ Desai J Mulay SR. Neutrophils and neutrophil extracellular traps drive necroinflammation in COVID-19. Cells. (2020) 9 (6):1383. doi: 10.3390/cells9061383 32498376 PMC7348784

[11] Lippi G Favaloro EJ. Epidemiology and predisposing factors of post-COVID venous thrombosis: A concise review. Semin Thromb Hemost. (2024) 50:271–4. doi: 10.1055/s-0043-1770051 37327881

[12] Zuin M Rigatelli G Zuliani G Roncon L. The risk of thrombosis after acute-COVID-19 infection. QJM. (2021) 114:619–20. doi: 10.1093/qjmed/hcab054 PMC7989150 33720351

[13] Constantinescu-Bercu A Kessler A de Groot R Dragunaite B Heightman M Hillman T. Analysis of thrombogenicity under flow reveals new insights into the prothrombotic state of patients with post-COVID syndrome. J Thromb Haemost. (2023) 21:94–100. doi: 10.1016/j.jtha.2022.10.013 36695401 PMC9773628

[14] Nehme M Ducrot A Salmon D Guessous I. Post-Covid: nouveautés 2022 et prochaines étapes [Post-Covid: 2022 updates and next steps. Rev Med Suisse. (2023) 19:160–6. doi: 10.53738/REVMED.2023.19.812.160 36723639

[15] Skendros P Mitsios A Chrysanthopoulou A Mastellos DC Metallidis S Rafailidis P. Complement and tissue factor-enriched neutrophil extracellular traps are key drivers in COVID-19 immunothrombosis. J Clin Invest. (2021) 131 (11):6151–57. doi: 10.1172/JCI141374 PMC7598040 32759504

[16] Zuo Y Yalavarthi S Shi H Gockman K Zuo M Madison JA. Neutrophil extracellular traps in COVID-19. JCI Insight. (2020) 5. doi: 10.1172/jci.insight.138999 PMC7308057 32329756

[17] Al-Kuraishy HM Al-Gareeb AI Al-Hussaniy HA Al-Harcan NAH Alexiou A Batiha GE. Neutrophil Extracellular Traps (NETs) and Covid-19: A new frontiers for therapeutic modality. Int Immunopharmacol. (2022) 104:108516. doi: 10.1016/j.intimp.2021.108516 35032828 PMC8733219

[18] Bautista-Becerril B Campi-Caballero R Sevilla-Fuentes S Hernández-Regino LM Hanono A Flores-Bustamante A. Immunothrombosis in COVID-19: implications of neutrophil extracellular traps. Biomolecules. (2021) 11:694. doi: 10.3390/biom11050694 34066385 PMC8148218

[19] Middleton EA He XY Denorme F Campbell RA Ng D Salvatore SP. Neutrophil extracellular traps (NETs) contribute to immunothrombosis in COVID-19 acute respiratory distress syndrome. Blood. (2020) 136:1169–79. doi: 10.1182/blood.2020007008 PMC7472714 32597954

[20] Bosteels C Neyt K Van Damme K De Beuckelaer A Vandecasteele S Knockaert S. Neutrophil extracellular traps in COVID-19. Bull Cancer. (2021). doi: 10.1016/j.bulcan.2021.01.005. ahead of print.

[21] (2024). Available online at: https://www.sanidad.gob.es/areas/alertasEmergenciasSanitarias/alertasActuales/nCov/situacionActual (Accessed August 14, 2024).

[22] htm World Health Organization World Health Organization. World health statistics 2023. In: Monitoring Health For the SDGs, Sustainable Development Goals. Geneva: WHO (2023). Licence: CC BY NC SA 3.0 IGO.

[23] Ding K Jiang W Xiong C Lei M. Turning point: A new global COVID-19 wave or a signal of the beginning of the end of the global COVID-19 pandemic? Immun Inflammation Dis. (2022) 10:e606. doi: 10.1002/iid3.606 PMC8962637 35349754

[24] Gao L Zheng C Shi Q Xiao K Wang L Liu Z. Evolving trend change during the COVID-19 pandemic. Front Public Health. (2022) 10:957265. doi: 10.3389/fpubh.2022.957265 36203708 PMC9531778

[25] Casado-Fernández G Corona M Torres M Saez AJ Ramos-Martín F Manzanares M. Sustained cytotoxic response of peripheral blood mononuclear cells from unvaccinated individuals admitted to the ICU due to critical COVID-19 is essential to avoid a fatal outcome. Int J Environ Res Public Health. (2023) 20:1947. doi: 10.3390/ijerph20031947 36767310 PMC9915056

[26] Merad M Blish CA Sallusto F Iwasaki A. The immunology and immunopathology of COVID-19. Science. (2022) 375:1122–7. doi: 10.1126/science.abm8108 PMC12828912 35271343

[27] Mohandas S Jagannathan P Henrich TJ Sherif ZA Bime C Quinlan E. RECOVER Mechanistic Pathways Task Force. Immune mechanisms underlying COVID-19 pathology and post-acute sequelae of SARS-CoV-2 infection (PASC). Elife. (2023) 12:e86014. doi: 10.7554/eLife.86014 37233729 PMC10219649

[28] Krinsky N Sizikov S Nissim S Dror A Sas A Prinz H. NETosis induction reflects COVID-19 severity and long COVID: insights from a 2-center patient cohort study in Israel. J Thromb Haemost. (2023) 21:2569–84. doi: 10.1016/j.jtha.2023.02.033 PMC10088279 37054916

[29] Sharma V Aden D Zaheer S Ranga S. The role of the formation of neutrophil extracellular traps (NETosis) in the pathophysiology and the complications of COVID-19: A review of the literatura. Saudi J Health Sci. (2023) 12:91–113. https://www.researchgate.net/publication/372880217 (Accessed August 14, 2024).

[30] Behzadifard M Soleimani M. NETosis and SARS-COV-2 infection-related autoimmunity: A narrative review. Thromb J. (2022) 20:13. doi: 10.1186/s12959-022-00375-1 35354492 PMC8965217

[31] de Diego C Lasierra AB López-Vergara L Torralba L Ruiz de Gopegui P Lahoz R. What is the actual relationship between neutrophil extracellular traps and COVID-19 severity? A longitudinal study. Respir Res. (2024) 25:48. doi: 10.1186/s12931-023-02650-9 38243237 PMC10797938

[32] Kharouf F Eviatar T Braun M Pokroy-Shapira E Brodavka M Zloof Y. A deep look into the storm: Israeli multi-center experience of coronavirus disease 2019 (COVID-19) in patients with autoimmune inflammatory rheumatic diseases before and after vaccinations. Front Immunol. (2023) 14:1064839. doi: 10.3389/fimmu.2023.1064839 36993961 PMC10040776

[33] (2024). Available online at: https://www.covid-19.seth.es/wp-content/uploads/2020/04/Recomendaciones-tromboprofilaxis-y-tratamiento-antitrombotico-pacientes-COVID-19.pd (Accessed August 14, 2024).

[34] Wei Q Mease PJ Chiorean M Iles-Shih L Matos WF Baumgartner A. Machine learning to understand risks for severe COVID-19 outcomes: a retrospective cohort study of immune-mediated inflammatory diseases, immunomodulatory medications, and comorbidities in a large US health-care system. Lancet Digit Health. (2024) 6:e309–22. doi: 10.1016/S2589-7500(24)00021-9 PMC11069366 38670740

[35] Pang L Liu Y Shen M Ye J Chen R Lan Z. Influence of aging on deterioration of patients with COVID-19. Aging (Albany NY). (2020) 12:26248–62. doi: 10.18632/aging.202136 PMC7803552 33232272

[36] Duan H Liang L Liu X Xie S Wang C. PARC/CCL18 is associated with inflammation, emphysema severity and application of inhaled corticosteroids in hospitalized COPD patients. Int J Chron Obstruct Pulmon Dis. (2021) 16:1287–97. doi: 10.2147/COPD.S304488 PMC8121623 34007168

[37] Giraldo P López de Frutos L Cebolla JJ. Biomarker combination is necessary for the assessment of Gaucher disease? Ann Transl Med. (2018) 6:S81. doi: 10.21037/atm.2018.10.69 30613656 PMC6291607

[38] Chupp GL Lee CG Jarjour N Shim YM Holm CT He S. A chitinase-like protein in the lung and circulation of patients with severe asthma. N Engl J Med. (2007) 357:2016–27. doi: 10.1056/NEJMoa073600 18003958

[39] Alshipli M Altaim TA Oglat AA Ahmed Alsenany S Khodrog O Hasan H. Predictive value of D-Dimer and thromboplastin time as coagulation indicators for COVID-19 patients. J Infect Dev Ctries. (2024) 18:666–71. doi: 10.3855/jidc.18593 38865388

[40] De Rop L Bos DA Stegeman I Holtman G Ochodo EA Spijker R. Cochrane COVID-19 Diagnostic Test Accuracy Group; Verbakel JY. Accuracy of routine laboratory tests to predict mortality and deterioration to severe or critical COVID-19 in people with SARS-CoV-2. Cochrane Database Syst Rev. (2024) 8:CD015050. doi: 10.1002/14651858.CD015050.pub2 39105481 PMC11301994

[41] Kozłowski P Leszczyńska A Ciepiela O. Long COVID definition, symptoms, risk factors, epidemiology and autoimmunity: A narrative review. Am J Med Open. (2024) 11:100068. doi: 10.1016/j.ajmo.2024.100068 39034937 PMC11256271

[42] Martinod K Wagner DD. Thrombosis: tangled up in NETs. Blood. (2014) 123:2768–76. doi: 10.1182/blood-2013-10-463646 PMC4007606 24366358

[43] Zhang S Huang G Yuan K Zhu Q Sheng H Yu R. Tanshinone IIA ameliorates chronic arthritis in mice by modulating neutrophil activities. Clin Exp Immunol. (2017) 190:29–39. doi: 10.1111/cei.12993 28542869 PMC5588760

[44] Kaneski CR Moore DF Ries M Zirzow GC Schiffmann R. Myeloperoxidase predicts risk of vasculopathic events in hemizgygous males with Fabry disease. Neurology. (2006) 67:2045–7. doi: 10.1212/01.wnl.0000247278.88077.09 PMC1950664 17159117

[45] Lee KH Cavanaugh L Leung H Yan F Ahmadi Z Chong BH. Quantification of NETs-associated markers by flow cytometry and serum assays in patients with thrombosis and sepsis. Int J Lab Hematol. (2018) 40:392–9. doi: 10.1111/ijlh.2018.40.issue-4 29520957

[46] Zonta YR Dezen ALO Della Coletta AM Yu KST Carvalho L dos Santos LA. Paracoccidioides brasiliensis releases a DNase-like protein that degrades NETs and allows for fungal escape. Front Cell Infect Microbiol. (2021) 10:1–13. doi: 10.3389/fcimb.2020.592022 PMC7902888 33643928

[47] Hakkim A Fürnrohr BG Amann K Laube B Abed UA Brinkmann V. Impairment of neutrophil extracellular trap degradation is associated with lupus nephritis. Proc Natl Acad Sci U S A. (2010) 107:9813–8. doi: 10.1073/pnas.0909927107 PMC2906830 20439745

[48] Jiménez-Alcázar M Rangaswamy C Panda R Bitterling J Simsek YJ Long AT. Host DNases prevent vascular occlusion by neutrophil extracellular traps. Science. (2017) 358:1202–6. doi: 10.1126/science.aam8897 29191910

[49] Zhao Z Liu X Shi S Li H Gao F Zhong X. Exogenous hydrogen sulfide protects from endothelial cell damage, platelet activation, and neutrophils extracellular traps formation in hyperhomocysteinemia rats. Exp Cell Res. (2018) 370:434–43. doi: 10.1016/j.yexcr.2018.07.007 29981342

[50] Wei M Tai G Gao Y Li N Huang B Zhou Y. Modified heparin inhibits P-selectin-mediated cell adhesion of human colon carcinoma cells to immobilized platelets under dynamic flow conditions. J Biol Chem. (2004) 279:29202–10. doi: 10.1074/jbc.M312951200 15133030

[51] Chalmers JD Moffitt KL Suarez-Cuartin G Sibila O Finch S Furrie E. Neutrophil elastase activity is associated with exacerbations and lung function decline in bronchiectasis. Am J Respir Crit Care Med. (2017) 195:1384–93. doi: 10.1164/rccm.201605-1027OC PMC5443898 27911604

[52] Garcia G Labrouche-Colomer S Duvignaud A Clequin E Dussiau C Trégouët DA. Impaired balance between neutrophil extracellular trap formation and degradation by DNases in COVID-19 disease. J Transl Med. (2024) 22:246. doi: 10.1186/s12967-024-05044-7 38454482 PMC10919029

