# Supplementary material for: Neutrophil extracellular traps and macrophage activation contibute to thrombosis and post-covid syndrome in SARS-CoV-2 infection
Source: Front Immunol. 2025 Feb 24;16:1507167. doi: 10.3389/fimmu.2025.1507167 (PMC11891236; doi:10.3389/fimmu.2025.1507167)
Supplement: Supplementary file 1 [file DataSheet1.pdf]

Supplementary Material: 4S Tables; 1S Figure

Table 1S: General characteristics

First wave at diagnosis (March 2020)				
**ID**	**Age**	**Gender**	**Comorbidities**	**Vaccination**
P01	66	M	H, L, C, A[1]	No
P02	68	M	D, L, C	No
P03	80	F	R, L, M, S, E, A[1]	No
P04	74	F	O,H, L, M, S, A[2]	No
P05	69	F	O, C, M, A[3]	No
P06	70	F	D,O	No
P06	70	F	Pneumonia	No
P07	66	F	D,O,R,H,M,E	No
P08	82	F	K	No
P09	75	M	R, L, H, A[4]	No
P10	72	F	H, M, A[5]	No
P11	68	M	H, C, M, S, N, K, A[6]	No
P12	70	F	D,R, H, M, A[1]	No
P13	65	M	D, L	No
P14	78	M	Pneumonia	No
P15	53	F	Pneumonia	No
P16	68	F	E, K, A[2]	No
P17	70	F	L, E, A[7]	No
P18	71	M	D, R, H, S, A[1]	No
P18	71	M	Pneumonia	No
P19	66	M	H, L, M, A[8]	No
P20	78	M	D, H, S, A[7]	No
P21	84	M	O,R,H, L, S	No
P22	82	M	Pneumonia	No
P23	80	M	R, L, S, K	No

P24	79	F	H, C, M, S, N, K, A[1]	No
P25	72	F	H, L, S, A[4]	No
P26	69	M	No	No
P27	70	M	No	No
P28	57	F	No	No
P29	63	M	No	No
P30	60	M	No	No
**Second wave at diagnosis (July 2021)**				
**ID**	**Age**	**Gender**	**Comorbidities**	**Vaccination**
P31	59	M	O, H, L	Yes
P32	45	F	A[9]	No
P33	25	M	M, A1	No
P34	33	M	O, H	Yes
P35	39	F		No
P36	32	M	M, A[8]	No
P37	49	M		No
P38	66	F	H, L	Yes
P39	59	M	D, H, L	No
P40	44	F	O, M	No
P41	43	M	A[1]	Yes
P42	52	F	A[6]	No
P43	42	F	O, H, A[1]	No
P44	42	M	M	Yes
P45	54	F	H, N	No
P46	39	F	O	No
P47	28	M	K, A[5]	No
P48	20	M	A[2,7]	No
P49	38	F	D, O, R, H, L, A[10]	Yes
P50	41	F	L, E	Yes
P51	34	F		No

P52	51	F	M	No
P53	29	F		No
P54	48	F		No
P55	22	F		No
P56	43	F	H	No
P57	35	M		No
P58	25	M		No
P59	41	M	L	No
P60	60	M	D, O, H, L, A[11]	Yes

D=Diabetes, O=Obesity, L=Dyslipemia, C= Hearth Disease, R= Respiratory disease (EPOC. Asma), M= mental dysfunction (Depression, Anxiety, Dementia), E= endocrinopathy, N=neoplasia, S= skeletal problems (Arthrosis, Osteoporosis), K=renal dysfunction, H=Arterial hypertension, A= Another/Other (gastrointestinal disturbances[1], non alcoholic liver disease[2], diziness[3], Parkinsonism[4], migraine[5], allergy[6], hard smoking[7], hemochromatosis[8], epilepsy[9], gall blader[10], adrenal insufficiency[11]).

Table2S. Analytical data at diagnosis and after 7 days in first and second wave

ID First wave	Hb g/dL	Leukocyt esx109/L	Neutroph ilsx109/L	Lymphocyt esx109/L	Platelet sx109/L	aPTT sec	PT sec	Fibrin ogen mg/dL	D-Dimer ng/mL	Ferriti n ng/mL	GGT U/L	AST U/L	ALT U/L	LDH U/L	PCR mg/dL	ProCal citonin ng/mL	IL-6 pg/mL
P01	16.2	6.8	5.2	0.7	158	31.8	12.8	3.1	505	780.5	52	81	119	185	1.04		
P01	16.0	5.4	4.0	1.1	119	30.4	12.9	5.9	618	1416.9	83	86	129	282	1.20	0.07	99.57
P02	10.1	7.8	3.2	2.7	430	31.9	13.8	6.0								0.09	
P02	12.7	14.2	12.9	0.3	300	23.1	12.0	5.3								0.24	
P03	13.5	2.1	1.6	0.4	102	26.1	13.1	>7	4144	445.0		58	32	248	2.29	0.11	31.73
P03	12.4	5.3	4.5	0.4	159	18.2	15.4	>7	602	577.5	52	16	14	185	9.50	0.09	33.14
P04	13.0	14.6	12.7	1.2	143	22.7	10.7	3.1	150	465.0	151	32	64	207	0.58	0.09	
P04	13.6	9.2	8.1	0.6	141	24.0	10.6	6.8			106	26	33	315			
P05	14.6	5.7	4.3	1.1	142	27.1	11.8	>7	703	757.1	34	42	48	371	3.86	0.08	44.29
P05	15.3	9.9	7.6	1.5	235	25.7	13.1	>7	1537	867.9	36	48	54	408	1.85	0.10	56.18
P06	8.9	5.6	4.5	0.4	196	40.0	28.4	5.3	208	78.4	12	16	9	200	10.94	0.16	55.19
P06	9.5	3.5	1.8	1.0	283	31.9	22.4	2.9	573	37.1	29	14	16	170	1.07	0.06	3.58
P07	12.4	4.1	2.6	1.2	183	35.3	12.4	3.9	2356	1260.6	84	64	84	429	6.49	0.15	137.7
P07	11.0	3.6	2.8	0.5	171	26.3	12.0	2.0	20316	665.1	238	166	293	464		0.06	65.39
P08	12.1	5.6	3.9	1.2	246	29.1	13.1	6.6	417	95.7	32	58	50	438	3.72	0.06	117.2
P08	12.8	8.0	5.2	1.7	271	27.3	13.8	3.5	434	70.2	35	22	41	216	0.10	0.03	66.36
P09	13.3	9.1	7.7	1.0	206	32.9	12.1	6.2	343	260.5	25	53	19	431	5.63	0.07	30.44
P09	13.5	20.3	19.3	0.6	303	25.3	13.6	6.5	7682	480.5	37	33	27	669	8.79	0.08	78.81
P10	13.3	6.8	5.9	0.4	155	30.1	12.3	4.2	402	88.0	10	16	14	166	0.48		

P10	12.5	6.3	5.0	0.6	138	26.8	9.6	8.0									
P11	14.3	6.0	4.5	1.2	95	28.9	11.4	5.8	1062	441.7	15	29	12	206	6.34	0.10	3.86
P11	14.1	11.1	9.4	1.2	145	22.7	12.0	>7	6088						12.09		
P12	12.7	5.5	4.1	1.2	99	19.3	12.6	6.7	5733	1472.8	31	88	65	347	3.86	0.22	39.71
P12	11.0	6.5	3.9	1.8	191	29.1	13.1	6.7	1256	706.5	39	39	76	205	1.31	0.12	20.66
P13	15.6	4.5	3.5	0.8	133	28.3	12.6	>7	278	1281.7		32	31	261	2.60	0.09	46.09
P13	14.6	5.9	4.6	0.9	353	23.7	10.6	>7	524	1281.7	137	41	41	271	2.60	0.09	46.09
P14	13.4	5.8	3.3	1.0	74	54.6	34.5	6.1	367	246.6	17	33	16	252	11.35	0.45	56.23
P14	13.1	6.2	3.7	0.8	86	39.6	15.5	8.0	364	1380.3	16	26	14	360	6.64	0.12	
P15	14.0	6.8	4.4	1.6	331	27.6	12.6	6.0	4533	82.4	22	36	24	313	8.40	0.08	12.58
P15	12.8	7.1	3.9	2.5	309	24.8	11.9	2.9	333	54.3	23	23	54	179	0.08	0.04	<1.5
P16	13.2	9.2	7.8	1.0	158	31.4	12.0	>7	366	597.3	109	34	21	381	8.24	0.06	108.4
P16	13.3	10.5	7.4	1.8	240	27.7	11.6	4.3	7675	1029.7	226	60	59	543		0.04	
P17	12.6	8.7	7.1	1.8	256	26.0	11.1	5.3	998	29.4	36	19	30	177	1.51	0.07	16.32
P17	12.0	10.1	8.9	1.6	252	26.6	11.4	6.8	718	41.4	46	19	27	195	5.01	0.08	31.47
P18	14.9	8.0	6.6	0.9	136	30.5	13.0	>7	430	2317.5	49	117	99	371	23.98	1.04	86.94
P18	13.8	3.7	1.8	1.2	261	27.5	12.4	5.2	549	1312.9	67	141	174	306	1.19	0.14	449.4
P19	14.6	8.2	5.4	1.7	147							15	11				
P19	15.1	7.4	3.9	2.2	203	30.5	12.9	8.0	1078	357.4	28	23	16	191	3.38	0.05	<1.5
P20	15.0	4.2	3.3	0.8	143	35.3	12.6	6.1	211	1029.7	236	156	217	379	8.62		
P20	12.2	7.3	6.4	0.5	192	32.6	11.6	6.3			358	98	125	404			
P21	11.8	9.6	8.6	0.5	247	37.8	51.7	7.8	685	1537.0	214	22	9	539	407.30	0.448	100.0
P21	11.2	10.4	8.8	0.8	244	25.8	14.5	5.4	733	1335.0	205	17	8	482	26.60	2.71	
P22	14.9	5.6	4.2	0.8	299	33.0	11.4	6.84	3215	577.0	35	25	20	489	27.70	0.033	
P22	14.9	8.2	4.8	2.4	262	32.5	10.5	6.67		688.0	96	33	46	366	3.20	0.039	
P23	8.2	8.7	5.7	1.8	230	34.6	14.0	6.5	4718	69.0	15	10	6	322	63.10	0.098	
P23	9.4	7.3	4.2	2.1	289	35.8	12.3	4.93	4564	63.0	22	14	9	382	23.80	0.058	

P24	13.0	7.7	4.9	1.8	392	28.1	12.6	6.22	3699	90.0				360	29.50	0.029	
P24	13.3	6.4	2.7	2.7	333	28.3	11.1	3.37	880	90.0	96	14	16	350	1.50	0.022	
P25	12.8	4.7	3.0	0.7	240	32.3	14.1	5.8	1073	130.0	47	72	52	305	18.10	0.145	
P25	14.8	4.9	2.0	2.3	130	29.5	14.0	6.37	541	230.0	68	54	71	263	9.0	0.02	
P26	15.1	7.0	3.9	1.7	154	27.7	13.1	5.5	298	104.0	18	33	28	173	1.01	0.16	5.62
P26	16.7	9.7	5.8	2.1	213	24.3	11.4	4.4	176	263.2	21	25	41	216	2.84	0.08	2.97
P27	11.7	4.1	2.6	0.6	190	30.3	14.1	6.2	1210	86.8		23	12	218	2.28	0.04	
P27	11.8	8.2	7.3	0.3	164	24.0	13.4	>7	1210	343.9	230	29	78	164	18.05	0.07	157.8
P28	10.9	5.6	3.2	1.1	122	40.5	32.6	6.4	728	319.9	27	18	6	174	11.14	0.09	48.3
P28	11.8	13.2	9.5	1.6	174	21.6	11.2	3.3	986	179.2	36	17	25	318	0.79	0.10	1.53
P29	14.0	3.3	1.9	0.9	102	29.6	12.5	5.6	718	763.4	73	38	31	212	2.63	0.11	33.86
P29	14.4	9.8	7.9	1.1	325	23.3	12.0	3.7	549	1106.9	80	42	94	320	0.61	0.03	132.4
P30	11.7	2.6	0.8	1.1	106	28.9	12.7	4.3	526	271.7	15	17	6	193	0.60	0.06	4.76
P30	12.2	4.0	1.7	0.7	132	23.4	13.1	5.7	938	484.3	28	25	11	265	2.55	0.13	

White rows corresponded to values at diagnosis and grey row corresponded to values 7 days after.

ID Second wave	Hb g/dL	Leukocytesx109/L	Neutrophilsx 109/L	Lymphocytesx 109/L	Plateletsx109/L	aPTT sec	PT sec	Fibrinogen mg/dL	D-Dimer ng/mL	Ferritin ng/mL	GGT U/L	AST U/L	ALT U/L	LDH U/L	PCR mg/dL	ProCalcitonin ng/mL	IL-6 pg/mL
P31	13.3	6.80	5.20	1.00	200	27.2	14.7	7	198	572.7	67	21	20	182	14.06	0.10	55.31
P31	12.0	9.80	8.00	1.20	274	26.6	13.9	7	528	579.1	84	15	21	155	8.58	0.07	
P32	12.0	3.30	2.20	0.80	206	28.7	14.5	5.6	1057	227.9	17	18	12	315	1.46	0.04	4.22
P32	12.7	6.90	4.20	2.10	219	30.2	12.8	4	476	175.8	17	15	10	265	0.24	0.03	
P33	13.4	8.30	5.60	1.60	289	26.6	13.8	7	513	674.8	70	41	48	319	3.13	0.09	48.29

P33	14.2	6.70	3.40	2.30	388	28.2	13.4	6.5	643	819.2	77	45	127	248	1.24	0.02	30.15
P34	14.2	6.20	4.50	1.00	163	32.2	13.7	7	490	1228.0	28	34	32	471	8.66	0.15	32.67
P34	14.9	10.20	7.60	1.60	304	26.8	12.4	4.4	507	1359.7	35	21	30	364	0.31	0.02	
P35	10.2	3.80	2.60	0.80	116	29.7	11.9	6.7	963	36.7	7	22	14	125	1.47	0.06	6.45
P35	11.7	5.00	4.20	0.70	132	27.7	11.9	7	1230	459.5	37	245	184	331	10.04	0.69	
P36	15.1	2.80	1.60	0.90	146	33.9	13.4	5.9	812	447.7	23	33	25	292	2.50	0.10	15.93
P36	15.3	7.50	6.30	0.80	186	34.3	11.4	5.1	353	988.7	27	71	58	356	0.68	0.05	
P37	16.6	5.50	3.40	1.50	156	31.5	12.0	4.4	318	282.2	56	44	49	269	0.95	0.09	8.25
P37	17.1	6.90	4.90	1.50	149	27.8	11.9	5.4	401	467.6	153	53	80	265	1.00	0.07	
P38	13.1	6.80	3.70	2.00	213	27.1	11.6	7	1238	203.8	41	25	25	228	1.75	0.07	
P38	12.0	7.50	5.90	0.90	165	28.5	13.4	7	1443	438.1	39	18	18	239	10.64	0.05	31.71
P39	12.0	12.60	11.60	0.50	292	26.3	14.7	6.3	1281	784.4	39	19	14	310	4.77	0.27	143.60
P39	13.4	8.00	5.30	1.70	281	24.5	14.3	3.1	4264	647.6	87	56	107	288	0.16	0.07	
P40	13.3	4.40	1.90	2.20	154	299.0	12.5	7	1523	266.1	23	36	53	170	3.90	0.02	3.41
P40	13.0	6.50	3.40	2.10	225	26.9	12.2	7	597	285.1	31	53	96	177	2.12	0.06	
P41	14.5	8.50	7.10	0.90	285	31.4	11.8	5.3	262	544.2	198	55	65	460	6.71	0.09	70.06
P41	15.3	16.40	12.20	2.80	457	25.3	12.9	3.3	418	552.3	431	57	177	580	0.41	0.03	
P42	11.7	6.10	4.50	1.00	397	36.0	11.1	4.6	605	714.7	76	ns	14	216	2.51	0.05	
P42	15.1	9.60	6.70	1.50	406	23.5	11.8	2.6	271	972.0	124	20	29	268	0.09	0.04	
P43	13.2	5.60	3.30	1.90	203	31.5	14.9	7	391	276.7	30	30	17	338	7.83	0.06	33.97
P43	13.7	13.50	8.70	3.40	354	29.1	16.1	6.1	423	283.7	34	22	36	303	1.39	0.02	
P44	14.3	4.70	7.00	1.30	209	35.6	11.6	6	505	1292.7	86	50	45	176	0.55	0.05	14.46

P44	13.6	12.70	10.30	1.20	286	31.0	12.6	7	572		91	40	54	123	1.78	0.11	
P45	12.5	2.30	1.60	0.40	101	37.6	12.3	7	374	1528.6	18	40	21	382	8.33	0.06	35.02
P45	13.4	3.90	3.40	0.90	196	34.0	11.8	7	217	1323.9	35	92	99	295	0.95	0.02	
P46	11.9	5.60	3.40	1.70	273	28.6	13.4	7	1096	462.5	76	46	36	250	22.12	0.13	98.73
P46	14.5	12.80	8.70	3.30	368	29.3	13.4	5.6	1095	309.4	79	23	38	244	1.58	0.03	
P47	11.7	3.60	1.80	1.40	115	30.6	12.1	4.8	765	791.0	24	33	31	243	0.39	0.10	0.39
P47	11.4	3.80	2.00	1.30	124	30.5	13.5	5.5	1276	975.1	31	31	28	264	1.62	0.08	1.62
P48	14.0	6.10	3.60	1.70	262	30.6	14.3	7	556	314.7	69	73	43	340	4.49	0.05	21.55
P48	15.1	11.00	9.50	0.80	420	26.6	12.2	6	202	301.8	100	109	119	294	1.29	0.02	
P49	13.1	4.70	2.80	1.50	183	31.5	1.02	7	779	232.1	31	50	39	285	8.67	0.05	20.80
P49	12.6	5.10	2.30	2.30	294	30.1	0.97	7	547	272.9	81	68	56	224	1.74	0.02	
P50	11.8	3.00	3.20	1.20	221	27.0	12.2	7	459	49.3	38	31	20	389	13.35	0.08	
P50	10.8	5.10	3.00	1.30	339	25.8	11.4	6.2	483	32.1	110	44	89	233	1.44	0.03	
P51	11.3	9.10	7.70	1.10	259	27.1	11.0	7	831	106.3	47	46	32	293	1.61	0.11	5.66
P51	10.3	10.30	7.30	2.10	377	24.3	12.2	7	1158	90.9	53	85	84	253	1.06	0.10	
P52	14.3	6.30	2.60	1.30	130	34.2	13.1	6.7	1146	610.1	31	37	21	440	2.79	0.03	4.37
P52	13.7	9.20	5.20	2.90	256	23.1	11.7	4.3	801	364.9	35	49	63	266	0.13	0.02	
P53	12.6	6.00	5.10	0.60	354	31.3	13.0	7	1952	1124.0	408	244	170	517	22.70	0.04	2.50
P53	12.3	8.10	6.60	1.00	576	26.8	12.1	7	615	401.2	481	84	224	286	1.01	0.02	
P54	13.3	1.90	1.10	0.70	190	30.5	30.5	6.7	599	18.3	13	27	13	246	6.98	0.05	2.78
P54	12.9	10.80	9.00	1.10	239	25.3	25.3	6.2	778	77.3	13	17	11	192	2.23	0.03	
P55	11.9	4.90	4.20	0.60	257	31.2	11.3	7	1357	85.6	19	27	13	268	14.67	0.95	64.71
P55	10.9	5.70	4.20	1.00	352	26.2	11.3	6.8	1619	109.3	32	22	12	231	1.41	0.49	3.51

P56	13.4	13.10	11.40	1.10	332	27.7	11.1	7	716	205.3	28	21	10	293	2.57	0.09	37.0
P56	13.1	19.50	15.70	2.40	568	25.2	12.1	4.4	503	148.0	39	26	29	258	0.20	0.06	
P57	15.9	9.10	6.70	1.70	238	38.0	15.5	7	259	542.2	66	34	29	503	23.83	0.36	
P57	15.3	11.40	7.80	2.40	454	28.8	13.1	5.1	326	424.5	64	38	125	316	1.61	0.05	
P58	14.3	3.60	2.20	1.10	175	33.0	13.7	7	422	847.8	26	56	49	352	5.31	0.09	
P58	14.5	4.80	2.50	1.80	302	31.6	13.3	6.4	289	685.5	26	51	80	226	0.96	0.02	
P59	14.2	6.90	4.90	1.50	264	31.1	12.9	7	488	416.1	40	46	40	345	12.54		
P59	13.5	7.20	4.90	1.60	339	32.6	14.2	7	591	428.1	45	39	51	307	11.59	0.10	
P60	13.7	8.70	7.20	0.90	334	28.0	13.6	7	11740	2583.8	127	57	36	787	36.18	0.37	13.94
P60	13.2	16.0	13.90	0.90	414	24.5	13.1	7	4606	2665.4	112	39	29	663	22.87	0.20	13.94

White rows corresponded to values at diagnosis and grey row corresponded to values 7 days after.

Table3S. Macrophage biomarkers and NET at diagnosis and after 7 days in first and second wave

ID<br>First wave	ChT activity nmol/mL/h	CCL18/PARC ng/mL	YKL-40 ng/mL	Free DNA ng/mcL	MPO ng/mL	NE ng/mL	DNase U/L	MRP ng/mL	P-SELng/mL
P01	70.70	277.0	126.59	0.245	363.0	16.0	1538.5	80.2	37.1
P01	51.07	274.0	34.52	0.225	165.0	13.0	1506.1	106.3	21.3
P02	76.22	310.0	62.35	1.00	493.0	31.2	1498.7	6089.0	23.0
P02	64.79	234.0	29.92	0.667	559.0	18.0	1497.7	583.1	55.5
P03	93.56	533.0	72.10	0.384	138.0	18.0	1477.8	579.0	9.7
P03	75.97	601.0	90.78	0.436	400.0	33.0	1476.9	784.9	14.7
P04	1.92	197.0	32.10	0.316	168.0	17.0	1614.5	48.3	25.9
P04	1.37	196.0	161.69	1.21	272.0	16.0	1561.4	1815.5	21.5
P05	42.59	271.0	213.81	0.349	186.0	21.0	1188.3	1007.9	12.8
P05	59.28	432.0	85.88	0.470	415.0	23.0	1302.3	797.9	23.7
P06	78.41	324.0	164.27	0.277	101.0	16.0	1504.1	353.5	26.4
P06	66.44	309.0	9.98	0.301	25.0	7.0	1405.5	153.3	18.1
P07	61.30	385.0	69.37	0.554	244.0	19.0	1330.2	1049.8	11.8
P07	45.78	288.0	12.05	1.14	338.0	16.0	1594.6	413.1	23.2
P08	70.24	370.0	3.81	0.426	368.0	20.0	1360.7	543.5	22.9
P08	71.76	205.0	213.81	0.348	123.0	12.0	1430.3	99.2	37.0
P09	89.29	452.0	16.14	0.442	201.0	29.0	1295.5	573.4	16.2
P09	98.54	668.0	32.80	0.601	108.0	17.1	1486.9	3284.0	58.6
P10	1.41	315.0	27.96	0.199	125.0	12.0	1361.6	216.0	12.9
P10	1.05	307.0	12.75	0.207	91.0	14.0	1271.0	159.5	16.3
P11	57.02	227.0	169.02	0.263	158.0	18.0	1422.2	528.3	10.8
P11	68.49	216.0	193.86	0.519	525.0	40.0	1440.5	1369.1	16.8

P12	64.14	613.0	227.90	1.07	325.0	42.6	1798.5	3954.0	84.0
P12	80.16	840.0	132.80	0.319	23.0	6.0	1474.0	534.5	10.5
P13	74.33	238.0	10.53	0.373	101.0	4.0	1463.0	209.0	18.1
P13	59.62	414.0	11.84	0.354	180.0	15.0	1675.1	671.3	12.8
P14	101.30	212.0	236.69	0.363	504.0	51.0	1201.2	841.7	15.2
P14	101.30	212.0	236.69	0.623	504.0	51.0	1201.2	841.7	14.9
P15	26.82	393.0	18.60	0.418	323.0	24.0	1215.2	1014.8	33.0
P15	27.46	62.0	19.98	1.24	233.0	18.0	1798.5	13.5	50.7
P16	92.58	121.0	47.20	0.248	168.0	21.0	1381.4	745.6	18.3
P16	75.57	84.0	2.35	0.922	302.0	13.2	1453.2	7030.6	44-6
P17	10.22	56.0	3.06	0.297	108.0	17.0	1057.5	299.0	16.9
P17	9.82	64.0	6.09	0.322	90.0	14.0	1297.4	207.9	14.4
P18	56.84	324.0	161.69	0.983	222.0	26.9	1632.6	1188.4	17.6
P18	36.72	307.0	47.20	0.568	85.0	17.0	1422.7	1023.4	17.5
P19	93.10	702.0	23.41	0.533	1133.0	147.0	1626.4	420.6	26.7
P19	141.55	532.0	227.90	0.344	85.0	19.0	1588.1	310.4	14.8
P20	16.37	108.0	69.37	0.262	67.5	23.2	1358.6	226.3	22.5
P20	20.04	78.0	10.53	1.25	198.26	26.3	1504.1	2210.3	20.6
P21	163.53	155.0	90.78	0.258	204.42	34.6	1201.2	3950.3	27.8
P21	149.02	71.0	164.27	0.256	74.42	9.6	1302.3	1877.0	23.7
P22	0.85	140.0	198.81	0.292	199.0	15.0	1632.6	346.7	26.4
P22	0.80	126.0	234.67	0.248	77.11	16.0	1588.1	39.2	24.2
P23	81.80	225.0	281.23	0.262	4.03	20.0	1474.0	105.2	30.8
P23	80.03	220.0	9.98	0.388	27.5	9.6	1476.9	341.3	32.3
P24	65.59	39.0	32.55	0.311	209.0	28.0	1626.4	1256.2	30.3
P24	61.78	231.0	72.10	0.390	25.57	16.0	1422.7	84.5	13.6
P25	71.11	420.0	132.80	0.598	66.34	20.0	1188.3	41.3	

P25	68.01	170.0	85.88	0.304	76.92	18.0	1477.8	24.2	28.5
P26	34.23	89.0	32.10	0.217	73.26	11.8	1405.5	165.2	24.0
P26	25.12	109.0	169.02	0.274	157.30	10.7	1330.2	141.3	50.0
P27	124.03	109.0	1.44	0.135	445.30	76.1	1463.0	5519.5	33.5
P27	140.82	46.0	12.05	0.213	137.5	24.2	1614.5	673.1	26.3
P28	35.88	75.0	126.59	0.248	110.0	44.0	1422.2	269.9	9.5
P28	36.57	54.0	11.84	0.319	146.15	23.7	1561.4	145.6	24.3
P29	49.92	17.0	27.96	0.301	90.38	17.8	1594.6	270.2	13.1
P29	59.84	22.0	9.42	0.388	90.96	20.0	1538.5	650.6	26.2
P30	95.52	85.0	3.06	0.195	132.88	23.6	1675.1	214,214	15.9
P30	108.22	80.0	34.52	0.243	233.65	26.0	1361.6	1062.8	17.9

White rows corresponded to values at diagnosis and grey row corresponded to values 7 days after.

ID Second wave	ChT activity nmol/mL/h	CCL18/PARC ng/mL	YKL-40 ng/mL	cfDNA ng/mcL	MPO ng/mL	NE ng/mL	DNAse U/L	MRP ng/mL	P-SELng/mL
P31	70.82	87.4	62.29	0.075	10.2	34.1	2603.6		9.5
P31	73.25	78.5	41.90	0.055	3.0	12.5	2919.5	293.5	15.2
P32	13.55	40.0	11.31	0.368	6.0	18.6	2484.0	304.2	15.5
P32	26.39	43.2	44.10	0.332	7.9	7.5	2534.0		16.0
P33	77.71	38,.2	15.96	0.237	17.7	65.4	2573.2	808.5	16.1
P33	52.87	28.0	10.37	0.118	12.1		2595.2		9.7
P34	55.55	89.3	37.41	0.603	469.6	70.4	2633.1	938.7	7.9
P34	39.04	56.8	17.41	0.372	189.6	69.4	2692.5	351.0	16.3
P35	22.23	12.9	30.71	0.266	2.7	16.3	2451.0		8.6
P35	13.94	24.3	23.30	0.703	82.4	45.0	2860.2	223.2	9.3
P36	20.09	20.0	31.87	0.381	25.5	61.8	2581.1	346.5	13.1

P36	18.38	20.1	97.50	0.410	129.6	137.9	2536.2	457.6	15.7
P37	66.56	45.5	17.58	0.338	158.0	46.3	2615.5	139.2	9.6
P37	81.45	46.0	28.75	0.443	288.0	84.3	2712.3	731.0	9.7
P38	35.31	66.4	36.25	0.840	84.6	18.3	2677.6	44.2	5.8
P38	48.48	86.6	53.48	0.282	217.1	95.3	2484.0	71.0	5.8
P39	49.91	44.5	60.44	0.556	143.8	141.9	2662.2	942.0	24.6
P39	74.74	39.5	23.59	0.201	4.6	38.1	2682.2		30.8
P40	22.07	34.6	10.50	0.262	16.5	51.4	2633.1		11.5
P40	23.47	61.9	8.36	0.080	5.9		2470.0		
P41	37.75	71.6	11.33	0.470	365.7	119.0	2568.1	687.6	18.5
P41	41.73	67.5	33.92	0.549	250.5	69.0	2919.5	799.8	37.1
P42	41.43	87.5	30.53	0.527	341.0	48.1	2412.2		14.6
P42	43.90	58.5	10.02	0.102	10391.0	24.3	2647.2		13.3
P43	26.77	60.0	35.26	0.571	80.2	45.6	2545.6	495.4	14.8
P43	20.93	72.0	12.64	0.262	21.1	57.2	2615.5	253.5	27.8
P44	99.45	76.9	75.00	0.444	147.7	96.3	2891.4	44.3	18.7
P44	32.67	49.0	18.07	0.932	84.6		2693.8		11.3
P45	21.21	21.2	52.14	0.585	258.2	67.5	2539.8	327.6	5.5
P45	6.51	21.9	26.69	0.772	363.0	73.6	2616.4	431.0	8.2
P46	51.02	22.8	14.01	0.415	55.2	62.0	2713.8	258.7	21.6
P46	72.35	34.1	16.87	0.387	43.2	26.9	2686.5	67.6	44.3
P47	42.91	16.0	13.63	0.238	24.4	17.6	2463.9	473.5	12.5
P47	45.85	15.3	19.14	0.246	27.0	52.0	2686.5	233.5	15.1
P48	33.35	63.6	14.55	0.478	156.8	34.3	2636.0	134.3	15.5
P48	27.60	65.6	8.59	0.465	154.3	79.7	2351.1	66.7	25.1
P49	33.98	75.1	298.30	0.303	6.3	8.8	2463.9		16.3
P49	34.99	98.2	182.41	0.396	5.5		2682.2	119.2	17.8

P50	13.85	59.0	15.26	0.364	4.5	20.2	2400.1	407.6	5.6
P50	23.36	39.8	7.64	0.248	16.3	38.3	2478.5	298.5	12.6
P51	30.43	36.5	31.78	0.347	143.5	15.6	2509.4		14.7
P51	20.34	66.5	6.15	0.212	94.6	24.8	2251.7		22.2
P52	89.09	61.9	7.14	0.416	274.9	78.9	2693.8	13.2	15.3
P52	84.86	71.1	8.21	0.445	244.3	57.5	2647.2		19.1
P53	15.52	67.5	20.80	1.57	312.7	58.1	2686.8	1907.0	50.4
P53	33.77	37.3	18.75	0.330	84.6	9.7	2603.6	697.6	54.8
P54	22.48	70.0	12.16	0.076	7.6	18.1	2351.1	271.7	8.6
P54	49.36	57.2	46.69	0.377	132.1	44.7	2478.5	275.4	19.3
P55	14.29	11.9	4.98	0.226	4.8		2713.8	138.5	
P55	17.36	13.3	4.53	0.124	5.3	22.9	2712.3	71.0	16.7
P56	16.49	16.7	114.82	0.431	564.3	160.5	2251.7	305.4	13.8
P56	34.50	16.8	65.08	0.401	313.8	37.5	2470.0	270.5	23.9
P57	18.66	106.3	27.76	0.501	244.3	148.3	2573.2	1582.0	20.3
P57	36.66	42.7	27.85	0.351	38.8	59.5	2595.2	228.7	24.2
P58	25.61	40.0	27.58	0.365	301.3	142.1	2590.1	122.1	9.1
P58	36.37	52.0	10.40	0.213	407.1	98.9	2686.8	591.0	13.6
P59	28.32	35.1	16.90	0.370	18.0		2568.1	908.5	7.2
P59	42.93	47.5	14.79	0.129	19.5	59.2	2636.0	358.5	17.9
P60	31.87	75.9	202.35	1.43	42.2	1.0	2451.0		3.8
P60	33.19	78.9	300.00	0.638	53.6		2534.0		

White rows corresponded to values at diagnosis and grey row corresponded to values 7 days after.

Table 4S. Laboratory normal ranges from healthy controls of analytical data, macrophages activation and NETs formation biomarkers

	Hb g/dL	Leukocytes $x10^9/L$	Neutrophils $x10^9/L$	Lymph $x10^9/L$	Platelets $x10^9/L$	aPTT sec	PT sec	Fibrinogen g/L	D-Dimer ng/mL	Ferritin ng/mL	GGT U/L	AST U/L	ALT U/L	LDH U/L	PCR mg/dL	ProCal ng/mL	IL-6 pg/mL
Controls range (Q1-Q3))	11.6-16.2	4.5-11.0	1.8-7.5	1.0-4.8	150-400	25.0-35.0	11.0-13.5	2.0-4.0	<500	15-300	5-40	10-34	5-60	125-220	<0.3	<0.5	<1.8

	ChT activity nmol/mL/h	CCL18/PARC ng/mL	YKL-40 ng/mL	cfDNA ng/mcL	MPO ng/mL	NE ng/mL	DNase U/L	MRP ng/mL	P-SELng/mL
Control range (Q1-Q3)	17-118	12-165	40.0- 73.3	0.33 – 0.56	28.65 – 58.60	4.35 – 7.98	849.34 – 1145.03	66.62 – 179.50	23.58 – 35.90

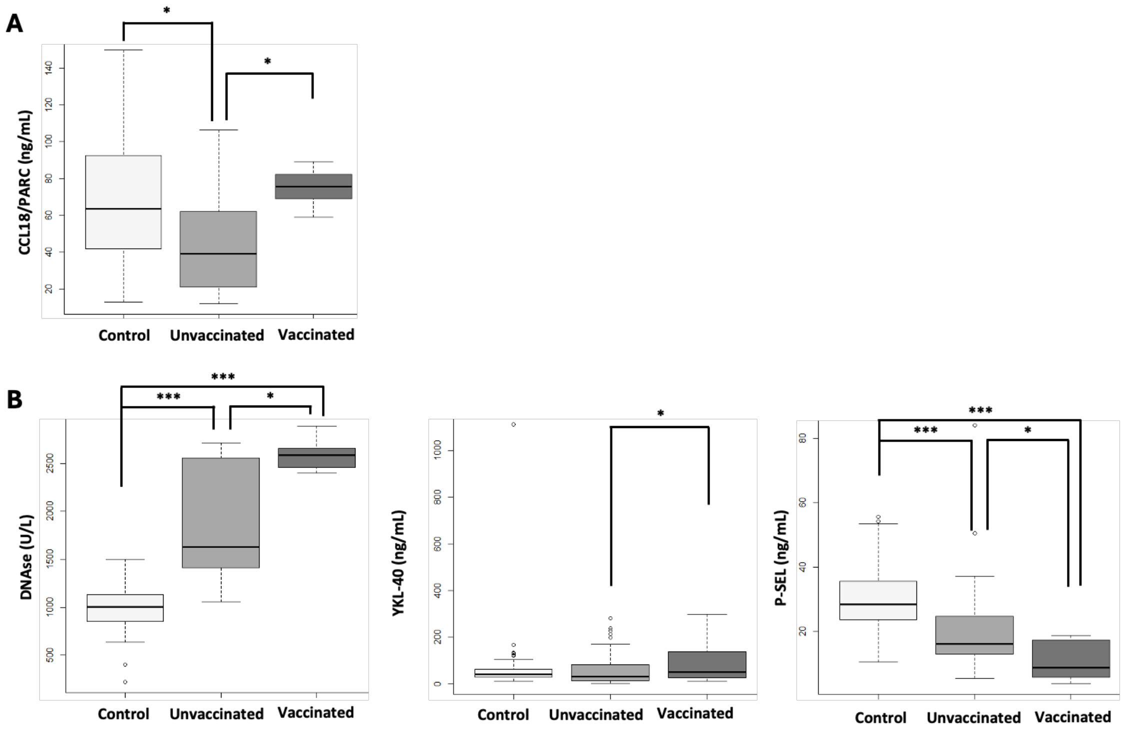

Figure 1S. Analysis of differences in macrophage activation and NETs formation biomarkers at the time of diagnosis in vaccinated and unvaccinated patients. **A**: In patients from second wave (N=30), an increase in the CCL18/PARC values in vaccinated (N=8) was observed compared to unvaccinated (N=22). **B:** In patients from both waves (N=60), a significant increase in DNase and YKL-40 was observed in vaccinated patients (N=8). In vaccinated patients (N=8) a significant decrease in P-SEL was observed. *p-value <0.05, *** p-value <0.0001
